# Mapping phenotypic performance and novel SNPs for cold tolerance in tomato (*Solanum lycopersicum*) genotypes through GWAS and population genetics

**DOI:** 10.1186/s12863-024-01190-5

**Published:** 2024-01-27

**Authors:** Labiba Riyaz Shah, Nazeer Ahmed, Khursheed Hussain, Sheikh Mansoor, Tamana Khan, Imran Khan, Sumati Narayan, Baseerat Afroza, Imtiyaz Murtaza, Asif Bashir Shikari, Basharat Bhat, Khalid Z. Masoodi

**Affiliations:** 1 Division of Vegetable Science, Sher-e-Kashmir University of Agricultural Sciences and Technology of Kashmir, Shalimar, Srinagar, Jammu and Kashmir 190025 India; 2 Transcriptomics Lab (K-Lab), Division of Plant Biotechnology, Sher-e-Kashmir University of Agricultural Sciences and Technology of Kashmir, Shalimar, Srinagar, Jammu and Kashmir 190025 India; 3 Division of Statistics, Sher-e-Kashmir University of Agricultural Sciences and Technology of Kashmir, Shalimar, Srinagar, Jammu and Kashmir 190025 India; 4 Division of Basic Sciences and Humanities, Sher-e-Kashmir University of Agricultural Sciences and Technology of Kashmir, Shalimar, Srinagar, Jammu and Kashmir 190025 India; 5 Division of Genetics and Breeding, Sher-e-Kashmir University of Agricultural Sciences and Technology of Kashmir, Jammu and Kashmir, Wadoora, Sopore, 193201 India; 6 NAHEP, IDP, Sher-e-Kashmir University of Agricultural Sciences and Technology of Kashmir, Shalimar, Srinagar, J&K 190025 India; 7 Department of Plant Resources and Environment, Jeju National University, Jeju, 63243 Republic of Korea

**Keywords:** Cold tolerance, Phenotype, Genome, Association studies, SNPs, Candidate gene

## Abstract

The cold stress susceptibility of tomato (*Solanum lycopersicum*) curtails its cultivation, with significant impact in temperate regions and on cropping seasons. To unravel genomic regions responsible for cold stress resilience, a diverse set of fifty genotypes encompassing cultivated, wild species, and landraces were genotyped using genotyping-by-sequencing. Over two years and six trials employing both early and late sowing, these lines were evaluated. Illumina-based next-generation sequencing produced up to 3 million reads per sample from individually sequenced library pools. The Tassel pipeline yielded 10,802 variants, subsequently filtered to 3,854 SNPs for genome-wide association analysis (GWAS). Employing clustering methods (population structure) via TASSEL, SNPhylo, and Kinship matrix, the fifty genotypes clustered into four distinct gene pools. The GWAS for cold tolerance in tomato integrated key traits including yield. Using six independent phenotypic datasets representing various environments, the study identified 4,517 significant marker-trait associations for cold tolerance traits. Notably, pivotal variations (> 10%) in cold stress tolerance, particularly proline content, were linked to marker-trait associations. Additionally, 5,727 significant marker-trait associations for yield and yield-related traits were unveiled, shedding light on fruit yield and directly associated attributes. The investigation pinpointed 685 candidate genes across all examined traits, including 60 genes associated with biological processes within these genomic regions. Remarkably, 7 out of the 60 genes were directly linked to abiotic stress tolerance, functioning as stress-responsive genes either directly or indirectly. The identified genes, particularly those associated with stress response, could hold the key to enhancing cold tolerance and overall crop productivity in tomato cultivation.

**Supplementary Information:**

The online version contains supplementary material available at 10.1186/s12863-024-01190-5.

## Introduction

Tomato due to its versatility and comparatively short genome (950 Mb), is commonly used as representative plant species for determining the genetic basis of complex traits, providing an ideal basis for current “-omics” research and genome guided breeding and has been employed both in conventional and molecular genetics. Nutrionally, the tomatoes are designated as ‘Protective Food’ as they are high in lycopene, ascorbic acid and beta-carotene. Consumption of lycopene-rich diets has been linked to a lower risk of numerous malignancies, including prostate cancer and coronary heart diseases (Karppi *et al*., 2009). Chen *et al*., (2015) found that increased lycopene consumption/circulating concentration is linked to a decreased possibility of prostate cancer and this red pigment (lycopene) is currently regarded as the “world’s most effective natural antioxidant”, making it a valuable ingredient in commercial therapeutic formulations [1, 2].

Tomatoes are extremely susceptible to chilling temperatures (0–12 °C), and many tomato-growing areas endure low (chilling) temperatures during the growing season, resulting in significant yield and colour reductions. Low temperature (cold stress) sensitivity of commercial varieties thus limits their geographic distribution, cultivation, growing period, or planting and harvesting times. Due to the prevalence of low temperatures, the number of cropping seasons is just limited to one only in temperate regions. Changes in ambient temperature have an unavoidable impact on biological processes. The rate of spontaneous and enzyme-catalysed chemical and physical reactions, the structure and molecular dynamics, and the strength of molecular contacts are all affected by temperature. Each of these side effects disrupts metabolism and cellular signal processing in some way. Due to direct suppression of metabolic activities as well as cold-induced osmosis (reduction of water uptake and cellular dehydration), the cold stress hinders plants from expressing their full genetic potential. Low temperature has a negative influence on tomato plant growth and development throughout its life cycle. It reduces germination [3], water status and photosynthesis affecting early phases of growth and development [4]. In addition, the reproductive development is also severely disrupted at all stages. Cold stress results in homeotic floral changes and reduced fruit set due to poor pollen germination, resulting in a significant drop in fruit production. Cultivars that are cold-tolerant can yield two or three cropping seasons each year. This would improve the efficiency with which existing processing capacity and fresh market availability could be improved and utilized [5].

In the past fruit yield, plant habit, adaptability to machine harvesting in processing cultivars and features linked to fruit appearance for the fresh market such as firmness, colour have been the focus of the majority of breeding studies. However in recent times due to climate change tomato breeding goals are more focused towards long-term productivity in changing environmental conditions. To broaden *Solanum lycopersicon’s* restricted genetic diversity, genetic variation from exotic germplasm collections is to be incorporated from other species like *Solanum pimpinellifolium, Solanum chilense*, *Solanum peruvianum*, *Solanum habrochaites* and *Solanum pennellii*. The first step includes studying the genetic diversity. Genomic revolution, during the last two decades, simplified understanding of the complex responses to biotic and abiotic stress in several crop plants [6, 7]. Since tomato species have spread across wide range of habitats of and are found in cold, dry, mesic and salty ecosystems at elevations above 3000 m MSL and therefore could be valuable for identifying and understanding the natural defence mechanism in *Solanum* species that allows them to deal with cold but not freezing conditions, known as cold acclimation. Plants use transcriptional, post-transcriptional and post-translational processes to modify their gene expression during cold acclimatization. Cold stress activates a variety of transduction pathways in response to environmental changes. Transcriptome studies of plants’ responses to abiotic stress revealed that the plant’s response to abiotic stress is mediated by many regulatory mechanisms.

Genome-wide association studies look at genetic variants across a variety’s genome to discover if any of them are linked to a trait [8] and has evolved as an useful tool for investigating and linking the vast volumes of data on genome sequence variation with observable behavioural differences. In GWAS the sample size is always large making it inaccessible for the researchers by being expensive but one of the approaches can be to use a star-like design by including geographically distant accessions (Arthur and Ashley, 2014). This maximizes the genetic variance within the sample (Li et al., 2010). Therefore, we used a core collection of diverse accessions collected from various agroclimatic regions to generate a basic and comprehensive dataset for cold tolerance traits apart from yield and yield related traits. QTL and GWAS has been conducted in tomato for various traits [9]. QTL studies for abiotic stresses have been conducted by Arms et al. (2015), Liu et al. (2016), Diouf et al. (2018) etc. in tomato. Also, three hundred eighty-eight suggestive association loci (including 126 significant loci) for ninety-two metabolic traits including nutrition and flavor-related loci were identified by genome-wide association study from various accessions in two different environments by Ye et al. (2019) [10]. The genetic architecture underlying tomato yield-related traits has been studied through GWAS. Based on ∼4.4 million single nucleotide polymorphisms obtained from diverse accessions, a comprehensive genome-wide association study for twenty-seven 27 agronomic traits in tomato have also been conducted (Ye et al. (2020) [11]. GWAS analysis of a collection of landraces and vintage was performed revealing that previously uncharacterized chromosomal regions were potentially involved in the expression of variable phenotypes by Rodriquez et al. But, a complete GWAS program with prime objective of cold tolerance has never been conducted. Therefore keeping in mind the lag this study was designed [12].

## Materials and methods

### Plant material

The experimental material comprised of fifty genotypes, used for identifying genomic regions and candidate genes for cold tolerance, yield and yield related traits collected from different agro- climatic regions. The list of genotypes is presented in Table 1. The collection included lines released as cold tolerant varieties viz., Pusa Sheetal, varieties grown and well acclimatized in cold stressed areas viz., Polish Tomato (heirloom variety from Poland; vigorous plants set fruit well even in cool weather), Sub Arctic Plenty (these tomatoes were developed in Alberta for prairie climates and set fruit under cold conditions), Coldset (an heirloom variety from Canada; the seeds withstand low temperatures), Black Plum (Russian heirloom variety, it is a fairly hardy cultivar and produces fruits in cooler temperatures) Stupice (it is Czechoslovakian-bred vine; it can blossom at cooler temperatures and can grow in climates as cold as those found in Alaska), Glacier (very cold tolerant and may survive a light frost also)., various species viz., WIR-3957 (*Solanum peruvianum)*, WIR-13,717 (*Solanum lycopersicum var cerasiforme)*, WIR-13,706 (*Solanum lycopersicum var cerasiforme)*, WIR-5032 (*Solanum chilense)*, WIR-13,708 (*Solanum lycopersicum var cerasiforme)*, IIHR-1939 (*Solanum pimpinellifolium)* and IIHR-2805 (*Solanum peruvianum)*, local landrace viz., Local-1 and breeding lines already identified as cold tolerant viz., IARI-2 and IARI-3.

**Table 1:** List of *Solanum* genotypes used in the study

S. No.	Genotypes	Species	Source	S. No.	Genotypes	Species	Source
1.	Shalimar-1	*S. lycopersicum*	SKUAST-K	26.	EC-620,402	*S. lycopersicum*	IIVR
2.	Shalimar-2	*S. lycopersicum*	SKUAST-K	27.	EC-620,438	*S. lycopersicum*	IIVR
3.	Black Plum	*S. lycopersicum*	SKUAST-K	28.	IIHR-1939	*S. pimpinellifolium*	IIHR
4.	Subarctic Plenty	*S. lycopersicum*	SKUAST-K	29.	IIHR-2201	*S. lycopersicum*	IIHR
5.	Polish tomato	*S. lycopersicum*	SKUAST-K	30.	EC-521,078	*S. pimpinellifolium*	IIVR
6.	Mortgage Lifter	*S. lycopersicum*	SKUAST-K	31.	IIHR-2805	*S. peruvianum*	IIHR
7.	Coldset	*S. lycopersicum*	SKUAST-K	32.	IARI-4	*S. lycopersicum*	IARI
8.	Glacier	*S. lycopersicum*	SKUAST-K	33.	IARI-7	*S. lycopersicum*	IARI
9.	Stupice	*S. lycopersicum*	SKUAST-K	34.	EC-145,057	*S. lycopersicum*	NBPGR
10.	H-88-78-1	*A derivative of S. habrochaites f. glabratum*	IIVR	35.	EC-164,334	*S. lycopersicum*	NBPGR
11.	IARI-2	*S. lycopersicum var cerasiforme*	IARI	36.	Local 2	*S. lycopersicum*	SKUAST-K
12.	EC-165,690	*S. lycopersicum var cerasiforme*	NBPGR	37.	EC-249,574	*S. lycopersicum*	NBPGR
13.	TOLCV-32	*S. lycopersicum*	IIVR	38.	EC-617,047	*S. lycopersicum*	NBPGR
14.	WIR-3957	*S. peruvianum*	IIVR	39.	EC-808,922	*S. lycopersicum*	NBPGR
15.	WIR-5032	*S. chilense*	IIVR	40.	EC-914,103	*S. lycopersicum*	NBPGR
16.	WIR-13,706	*S. lycopersicum var cerasiforme*	IIVR	41.	S-22	*S. lycopersicum*	SKUAST-K
17.	WIR-13,717	*S. lycopersicum var cerasiforme*	IIVR	42.	EC-914,106	*S. lycopersicum*	NBPGR
18.	VRT-01	*S. lycopersicum*	IIVR	43.	WIR-13,708	*S. lycopersicum*	IIVR
19.	Local 1	*S. lycopersicum*	SKUAST-K	44.	EC-914,108	*S. lycopersicum*	NBPGR
20.	TOLCV-16	*S. lycopersicum*	IIVR	45.	Pusa Sheetal	*S. lycopersicum*	NBPGR
21.	IARI-3	*S. lycopersicum*	IARI	46.	EC-914,112	*S. lycopersicum*	NBPGR
22.	EC-521,047	*S. lycopersicum*	IIVR	47.	EC-914,113	*S. lycopersicum*	NBPGR
23.	EC-528,360	*S. pimpinellifolium*	IIVR	48.	Roma	*S. lycopersicum*	SKUAST-K
24.	EC-528,372	*S. lycopersicum var cerasiforme*	IIVR	49.	EC-914,091	*S. lycopersicum*	NBPGR
25.	IARI-1	*S. lycopersicum*	IARI	50.	EC-914,092	*S. lycopersicum*	NBPGR

### Phenotyping

In case of tomato, the optimal temperature 15^°^C to 30^°^C. Tomatoes are sensitive to cold stress and it has a negative impact on tomato plant growth and development throughout its life cycle. Cold temperatures decrease germination early in development, the reproductive development is severely disrupted at all stages, Cold stress results in homeotic floral changes and reduced fruit set due to poor pollen germination and Furthermore, the low temperature causes tomatoes to fail to ripen normally. Early and late sowing a simple and effective technique for both germination and reproductive stage was adopted for phenotyping the germplasm along with planting at optimal periods. The fifty lines were evaluated for two consecutive years 2019 and 2020 under normal sown environment (E1: Field, normal sown (2019); E2: Polyhouse sown (2019); E3: Field, early sown (2020); E4: Field, normal sown (2020); E5: Field, late sown (2020); E6: Polyhouse sown (2020).

Experiments were conducted at experimental farm of the Division of Vegetable Science, SKUAST-K, Shalimar (34^o^ N latitude and 74.89^o^ E longitude, 1685 m above MSL). The population was evaluated in randomized block design. The spacing followed was 60 cm × 45 cm. All the individuals of the population were apportioned into a total of three blocks along replications. The maximum, minimum, and mean temperatures were recorded daily during the entire cropping season for both years. The mapping population was phenotyped for yield and yield related traits viz., days to emergence, seedling length at transplant (cm), number of flowers per truss, number of days to first fruit set, number of fruits per truss, number of days to first harvest, average fruit weight (g), fruit shape index, number of fruits per plant, fruit yield per plant (kg), number of primary branches, plant height (cm) and duration of harvest and cold tolerance traits viz., pollen viability (% (fluorescent microscope (LMI microscope ABE-UK)), malondialdehyde content (nmol gfw^−1^ [13]), proline content (µmol gfw^−1^ [14]), total leaf chlorophyll content (mg 100^−1^g [15]), ascorbic acid (mg 100^−1^g A.O.A.C (1984)), lycopene content (mg 100^−1^g [16]), total phenols (mg 100^−1^g (Malick and Singh (1980) and total soluble sugars (mg 100^−1^g [17]).

### Analysis of variance, best linear unbiased prediction (BLUP), and heritability

The ANOVA for the genotypes was performed using Metan: An R package [18], for individual environments using the mixed model analysis. For each trait and environment, the analysis was performed considering entry and block (nested within replication) as random effects and replication as fixed effects.

Broad-sense heritability was calculated as H_2_ = Vg/Vp.

In GAPIT-R, the best linear unbiased predictors (BLUPs) of each genotype were calculated for each environment. The calculated BLUPs were then used in the GWAS analysis.

### DNA extraction, genotyping and single-nucleotide polymorphism calling

DNA from 50 genotypes was isolated from leaf samples by using Xcelgen Plant gDNA Isolation Kit (XG2611-01) as per the protocol described. The DNA was eluted in 50 µl Nuclease-Free Water. The samples were quantified using Qubit Fluorometer. For determining A260/280 ratio, 1 µl of sample was loaded in Nanodrop8000 spectrophotometer. The quality of samples was checked in 0.8% Agarose gel electrophoresis along with Hind III marker for the presence of intact bands. The DNA from samples were digested using A*pe*K1 enzyme and the GBS library was prepared from the digested DNA fragments by ligating adaptors specific to the cut-site. The library pool was analyzed in Bioanalyzer 2100 (Agilent Technologies) using High Sensitivity (HS) DNA chip and then sequenced independently on Illumina platform to generate up to ~ 3 million reads/ sample. These barcodes tagged fastq files were analysed. TASSEL5 GBSv2 pipeline was used for identification of tags at cut sites and SNPs located across reference genome. Tomato reference genome (S_lycopersicum_chromosomes.4.00.fa.gz) employed was downloaded from solgenomics /(ftp://ftp.solgenomics.net/tomato_genome/assembly/build_4.00/). The fastq files were then aligned against the tomato reference genome using the Bowtie2 tool version 2.2.9. The .sam file created from the Bowtie2 aligner program was used through SAMToGBSdbPlugin.The alignment file was then processed by using the GBSv2 analysis pipeline for SNP calling and genotyping.

### Population structure, kinship and linkage decay (LD) analysis

STRUCTURE software (v.2.3.4). was used to assess the population genetic structure among 50 tomato genotypes [19] with admixture model. Population structure was estimated based on total SNP loci (3854 SNPs) and K from 1 to 10 with 10 independent runs for each K. To determine the probable number of clusters based on genotypes, the software parameters were set to 5000 burnin and 50,000 MCMC (Markov Chain Monte Carlo) iteration. Structure output was then subjected to structure harvester for identification of effective number of clusters using the Evanno test implement in STRUCTURE HARVESTER (Earl 2012). The Principal component analysis was obtained using TASSEL (5.0) for determination of percentage of variation explained by top three principal components. For phylogenetic analysis the SNP data was imported to SNPhylo with following parameters;-m 0.05 –a 478 –A-M 0.5-b-B 192; where m is the minor allele frequency; a is the total number of autosomes, b is Performs; M Missing rate. Tassel was used for generation of Kinship matrix and to know the relatedness among 50 tomato genotypes based on shared alleles among individuals. Linkage Disequilibrium (LD) was estimated using TASSEL (5.0) and LD curve was fitting using a nonlinear model as described by [20].

### Genome wide association analysis (GWAS)

The Genome-wide association analysis involves regressing each SNP separately on a given trait, adjusted for various confounding variables. After removing four controls, the genotypes were processed for GWAS analysis. TASSEL and GAPIT was used for GWAS analyses. SNP-trait association analysis was performed using Mixed Linear Model (MLM) [21] implemented in TASSEL that include correction for both population structure and kinship. GAPIT was also used for GWAS analysis, which is a Genome Association and Prediction Integrated Tool. GAPIT is a package that is run in the R software environment. GAPIT’s MLM (Mixed Linear Model) was used for GWAS analysis. Six phenotypic data sets representing each of six environments were used independently for genome wide association study. After GWAS analysis, SNPs showing association with particular trait at P-value < = 0.05 were considered as significant SNPs. The qqman R package [22] was used to plot Manhattan and quantile-quantile (QQ) plots. A 5% significance level was used to identify SNPs significantly associated with trait.

### Identification of candidate genes

Functional annotation of the predicted underlying genes with significantly associated SNPs was performed using the *Solanum lycopersicum* SL4.0 genome browser. Nearest genes located upstream or downstream of the significant markers were considered. Gene models were blasted against Tomato Genome Proteins (ITAG release 4.0) to determine the gene annotation.

## Results

### Phenotypic performance and genetic variability

The Analysis of variance performed on the traits using environment (Y), genotype (G) and genotype x environment (G x Y) interactions as effects of the model as presented in Table 2 revealed highly significant differences among all the genotypes under study for all traits and environments except for the trait fruit shape index thereby indicating a good amount of variability in the present material. The perusal of Table 3 revealed that mean performance of genotypes for various traits under different environments showed large variation and also the range was high for almost all the traits under study indicating that wide variation existed in the population.

**Table 2:** Phenotypic Performance and Genetic Variability using environment (Y), genotype (G) and genotype x environment (G x Y) interactions

		ENVIRONMENT			GENOTYPE			GENOTYPE: ENVIRONMENT		
	Traits	DF	MSS	F. Value	DF	MSS	F. Value	DF	MSS	F. Value
1	Days to emergence	5.00	530.89	241.64	49.00	17.77***	64.91	245.00	6.031	22.02
2	Seedling length	5.00	78.963	6.6	49.00	119.71***	358.2	245.00	46.108	138.0
3	Plant height	5.00	354905.9	4954.97	49.00	42067.5***	2387.05	245.00	6141.5	348.49
4	No of primary branches	5.00	82.024	23.99	49.00	15.337***	75.17	245.00	1.660	8.13
5	No of flowers/truss	5.00	156.488	37.3	49.00	144.09***	494.5	245.00	6.700	23.0
6	No of days to fruit set	5.00	15400.37	1369.8	49.00	243.74***	451.8	245.00	120.53	223.4
7	No of days to first harvest	5.00	5055.808	373.8	49.000	170.30***	361.4	245.000	188.197	399.4
8	No of fruits/truss	5.00	46.841	11.2	49.00	61.94***	264.6	245.00	7.485	32.0
9	Fruit size index	5.00	0.12046	11.78	49.00	0.52***	321.59	245.000	0.07793	48.19
10	Average Fruit weight	5.00	18042.0	1352.60	49.00	20858.8***	13947.86	245.0	851.8	569.61
11	Fruit yield	5.00	15.32	3.61	49.00	8.46***	2.02	245.00	5.56	1.33
12	No of fruits/plant	5.00	28074.3	747.07	49.00	37.6***	2.69	245.00	5173.1	370.08
13	Duration of harvest	5.00	2.94e + 05	6286.0	49.00	2.21e + 03***	1262.4	245.00	1.16e + 03	661.9
14	Pollen Viability	5.00	3378.97	123.36	49.00	3492.50***	463.94	245.00	641.34	85.20
15	Proline content	5.00	0.7975	9.643	49.00	0.3657***	4.194	245.00	0.1287	1.476
16	Lycopene	5.00	347.7557	647.3	49.00	135.48***	3324.2	245.00	6.0281	147.9
17	Total soluble sugars	5.00	3.81883	139.80	49.00	0.86***	305.98	245.000	0.23941	84.97
18	Total phenols	5.00	12,801,919	7628.9	49.00	4,179,959***	2745.9	245.00	1,323,887	869.7
19	Ascorbic acid	5.00	137.546	72.9	49.00	1264.17***	7355.6	245.000	58.967	343.1
20	Malondial dehyde content	5.00	18.9505	58.20	49.00	135.63***	2851.49	245.0	13.4041	281.79
21	Total leaf chlorophyll content	5.00	2049.915	1023.4	49.00	762.65***	4786.2	245.0	59.419	372.9

**Table 3:** Environment wise estimates of mean and range for different traits in tomato (*Solanum *spp.)

	**ENV**	**DTE**	**SL(cm)**	**NOFPS**	**NODTFS**	**NOFPT**	**NODTFH**	**AFW (g)**	**FSI (cm**^**2**^**)**	**NOFPP**	**FYP (kg)**	
Mean	E1	8.73	17.6	4.86	37.3	4.04	91.9	38.3	0.94	52.7	1.08	
	E2	11.00	18.7	6.91	26.0	4.93	81.7	56.9	0.92	47.5	1.56	
	E3	11.70	18.2	4.82	47.9	3.87	94.3	36.4	1.01	57.0	1.06	
	E4	7.99	16.9	4.68	36.7	3.96	91.3	32.4	0.94	72.8	1.30	
	E5	7.46	18.1	5.76	48.6	4.67	85.8	33.9	0.95	31.9	0.93	
	E6	11.40	18.8	6.82	25.2	5.18	80.2	55.3	0.93	44.5	1.75	
Range	E1	5.50-16.40	7.33-25.94	2.66-12.73	24.74-54.33	2.33-9.00	86.00-95.66	0.95-127.46	0.53-1.46	3.33-210.66	0.19-4.14	
	E2	7.66-14.66	7.66-28.00	3.33-25.66	22.66-47.66	2.33-17.33	81.66-91.66	2.45-318.00	0.50-1.25	4.00-434.66	0.03-4.90	
	E3	10.33-15.66	7.55-27.49	2.33-14.66	36.66-89.33	1.66-9.00	83.66-134.33	0.96-162.19	0.58-1.62	5.66-357.33	0.14-3.33	
	E4	5.00-14.66	6.28-23.78	2.33-19.66	27.33-54.33	2.00-17.66	85.66-96.66	1.32-148.49	0.47-2.17	4.66-364.66	0.19-3.98	
	E5	4.66-9.33	7.14-24.98	2.00-20.33	33.00-83.00	1.66-18.33	81.33-96.33	0.87-120.09	0.65-1.45	3.00-335.00	0.06-2.11	
	E6	9.66-15.66	6.77-24.52	3.66-24.66	22.33-43.33	3.33-18.33	81.66-87.66	1.42-319.89	0.51-1.41	3.20-402.33	0.08-5.73	
	**ENV**	**NPP**	**PH (cm)**	**DOH**	**PV (%)**	**MDA(nmol/gfw)**	**PC(µmol/g)**	**TCC(mg/100g)**	**AA(mg/100g)**	**LYC (mg/100g)**	**TP(mg/100g)**	**TSS(mg/100g)**
Mean	E1	3.50	118.57	113.48	47.9	6.43	0.68	13.6	15.3	6.41	1595.16	1.34
	E2	4.33	191.30	196.28	58.0	6.93	0.80	19.2	17.1	8.65	2245.19	1.07
	E3	3.29	94.4	140.10	49.5	6.80	0.70	12.1	15.7	6.71	1730.15	1.25
	E4	2.95	90.7	111.58	47.8	6.78	0.67	13.9	15.5	6.63	1651.14	1.39
	E5	2.52	87.0	95.5	47.8	5.99	0.67	9.63	14.7	4.63	1718.22	1.38
	E6	4.36	187.27	195.27	56.7	6.84	0.84	18.4	17.0	8.64	2217.19	1.02
Range	E1	2.00-5.33	33.33-198.33	94.88-133.33	27.11-92.53	2.03-17.00	0.49-0.89	4.22-41.00	7.22-43.22	0.89-13.15	154.23-3382.70	0.41-2.91
	E2	2.33-10.33	48.63-397.00	160.66-217.66	14.61-93.03	1.11-13.11	0.32-1.24	7.19-47.22	7.17-45.17	0.81-14.17	871-4122.00	0.76-1.36
	E3	1.66-8.33	42.83-165.05	115.66-167.66	18.23-92.00	0.76-17.41	0.44-1.49	3.22-36.10	7.77-41.27	0.01-13.70	507.20-3893.00	0.78-1.99
	E4	1.66-5.66	48.18-137.33	92.66-133.33	22.21-93.84	0.12-16.00	0.51-0.87	3.23-40.97	7.54-40.14	0.88-16.70	151.20-3255.20	0.44-2.94
	E5	1.33-4.00	42.15-156.92	85.66-120.33	10.13-92.67	0.26-17.16	0.49-0.89	3.22-27.22	6.19-42.17	0.41-10.22	211.20-3407.20	0.77-2.00
	E6	2.66-9.00	53.35-394.16	172-214.66	13.43-95.25	2.06-13.22	0.30-1.22	5.72-54.50	7.88-46.22	0.79-16.24	451-4050.00	0.78-1.39

E1: Field, normal sown (2019); E2: Polyhouse sown (2019); E3: Field, early sown (2020); E4: Field, normal sown (2020); E5: Field, late sown (2020); E6: Polyhouse sown (2020)

*DTE* Days to emergence, *SL* Seedling length at transplant, *NOFPS* Number of flowers per truss, *NODTFS* Number of days to first fruit set, *NOFPT* Number of fruits per truss, *NODTFH* Number of days to first harvest, *AFW* Average fruit weight, *FSI* Fruit shape index, *NOFPP* Number of fruits per plant *FYP* Fruit yield per plant, *NPP* Number of primary branches, *PH* Plant height, *DOH* Duration of harvest, *PV* Pollen viability, *MDA* Malondialdehyde, *PC* Proline content, *TCC* Total leaf chlorophyll content, *AA* Ascorbic acid, *LYC* Lycopene content, *TP* Total phenols, *TSS* Total soluble sugars

### Population structure, distribution, principal component analysis, kinship matrix and linkage disequilibrium (LD)

**Fig. 1:**
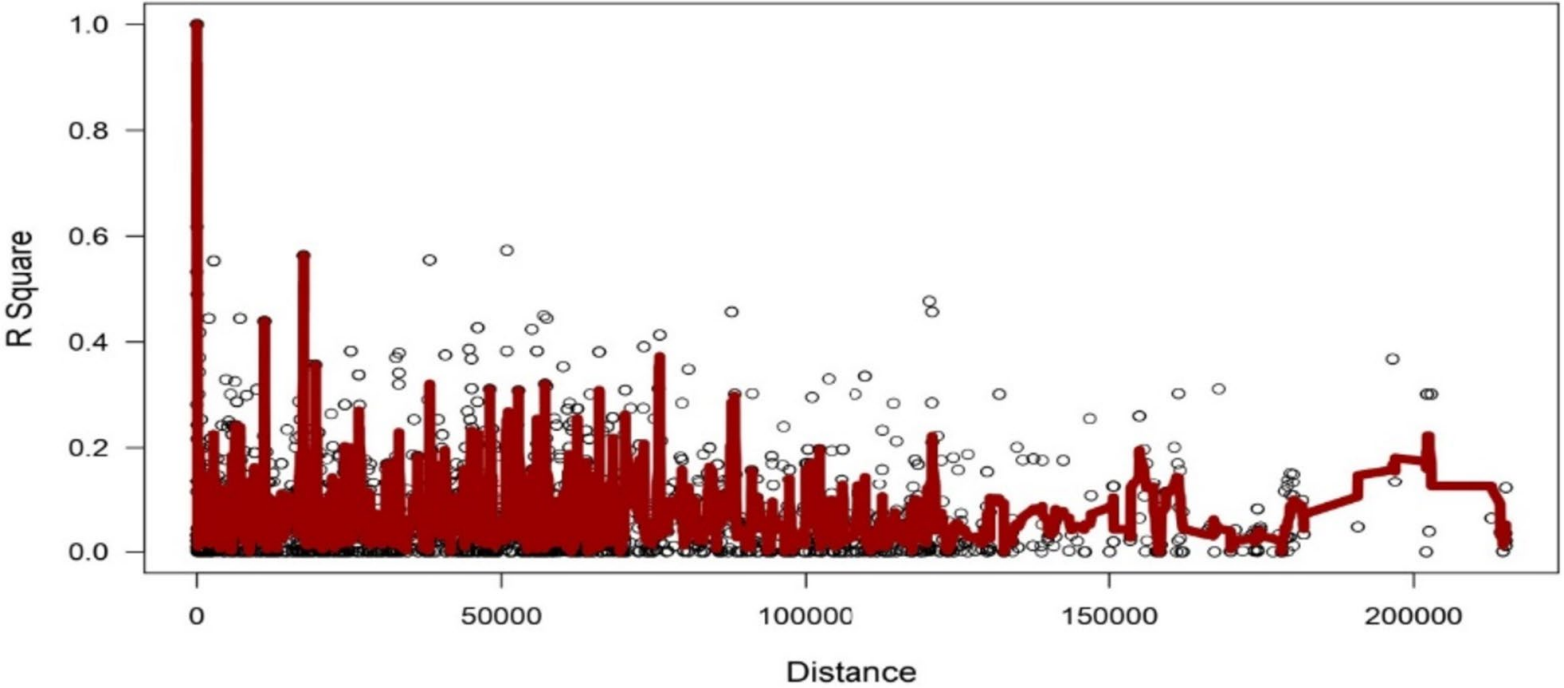
Linkage disequilibrium plot

Three million reads/sample were generated by sequencing using on Illumina platform. The raw sequence data were filtered to remove low-quality bases, adapter contamination and uncalled bases to produce high-quality sequence data. Then Tassel pipeline resulted in generation of 10,802 SNPSs after SNPS calling. After applying various quality-filtering parameters (MAF > = 0.05, MAC > = 10, Missing Data < = 50%) 3854 SNPSs were retained for downstream analysis. of 10,802 variants. Linkage decay was observed after 1.0 Mb distance (Fig. 1) that has a practical significance of identifying significant trait association even with a smaller number of markers.

**Fig. 2:**
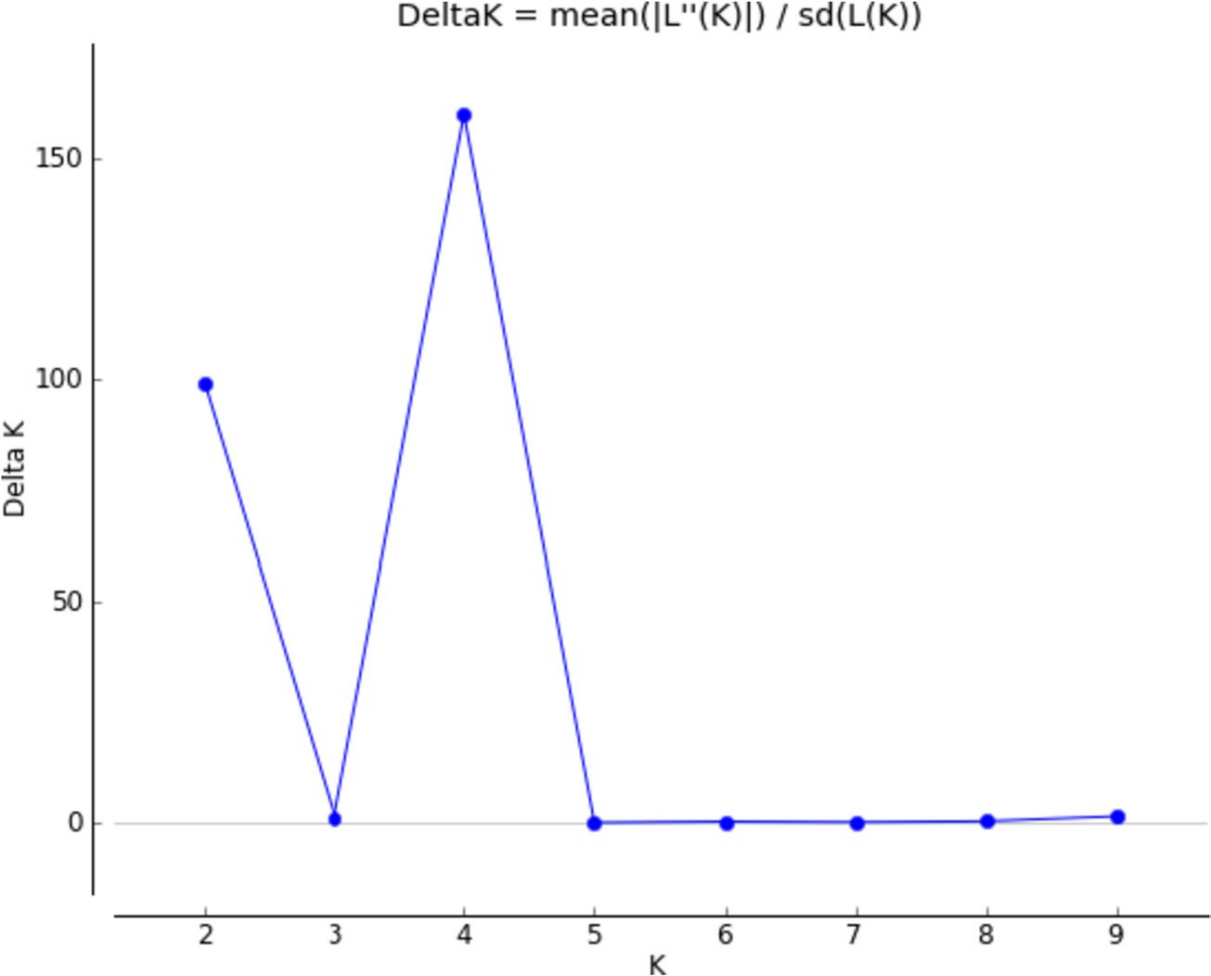
Plot showing the ΔK values for cluster size K = 1 to K = 10. The estimation of ΔK is performed at 10 independent runs burnin = 50,000 and MCMC = 100,000. Optimum cluster was obtained at K4

Using SNPs genotype file, the population structure within 50 genotypes was investigated. The ΔK method was used to infer the correct number of subpopulations. The ΔK method takes the rate of change of the mean probability values (LnP) of each subpopulation into consideration. The structural analysis led to the identification of four (K = 4) genetically distinct subpopulations. The rate of change was maximum (159.83) at K = 4 (Fig. 2); therefore, we considered four subpopulations in our population of 50 tomato genotypes.

**Fig. 3:**
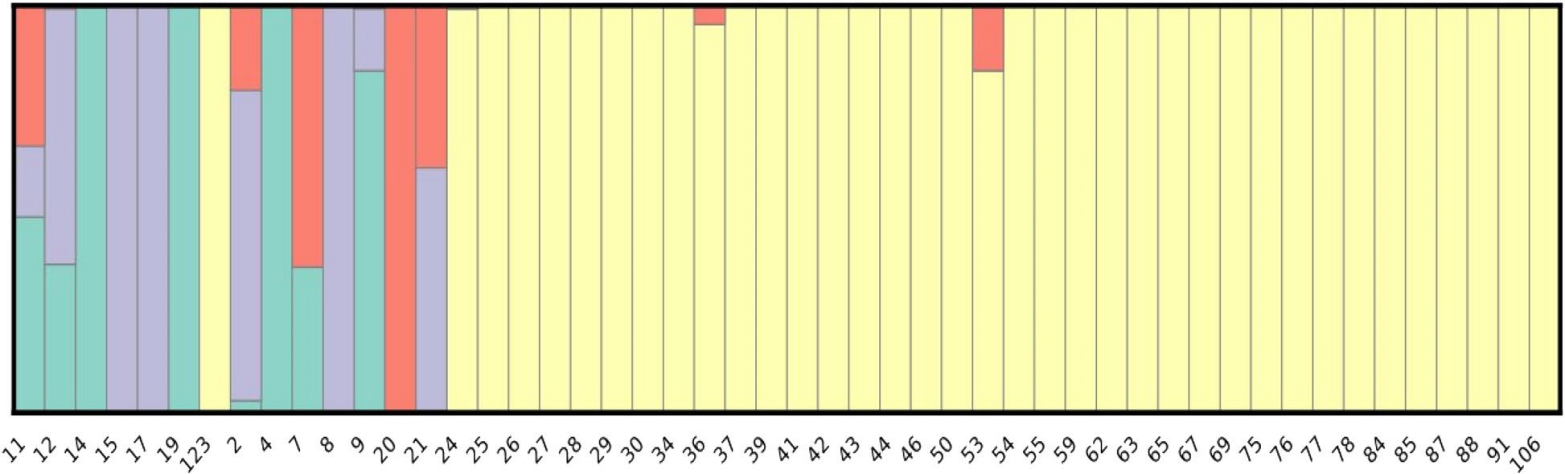
Bar Plots for K = 4 showing the population structure and genetic diversity present in each sample with their admixtures from 4 populations. The admixtures for each population in each sample shows the total proportion of each genotype present in the samples and their diversity

For the visualization of population structure, admixture analysis and population distribution at optimum K value of 4, bar plot was generated (Fig. 3). Then, based on the admixture coefficient obtained from STRUCTURE, number of samples falling under each population was determined. Genotypes with membership probabilities higher than 0.6 were assigned to one of the subpopulations (Table 4) whereas remaining were considered as admixed.

**Table 4:** Population assigned based on admixture coefficient

S. No.	Genotype	P1	P2	P3	P4	Population
1	EC-620,402	0.481	0	0.174	0.345	AD
2	TOLCV-16	0.364	0.001	0.627	0.008	P3
3	WIR-3957	0.996	0	0.001	0.003	P1
4	IARI-2	0	0	1	0	P3
5	EC-617,047	0.001	0	0.999	0	P3
6	COLDSET	0.997	0	0.002	0.001	P1
7	ROMA	0	0.999	0	0	P2
8	WIR-13,717	0.03	0	0.762	0.207	P3
9	H-88-78-1	1	0	0	0	P1
10	VRT-01	0.357	0	0.001	0.643	P4
11	WIR-5032	0.003	0	0.997	0	P3
12	EC-521,047	0.84	0	0.152	0.007	P1
13	EC-165,690	0	0	0	0.999	P4
14	GLACIER	0.002	0.002	0.597	0.398	AD
15	EC-249,574	0.001	0.99	0.008	0.001	P2
16	IARI-1	0	0.999	0	0	P2
17	EC-620,438	0	0.999	0	0	P2
18	EC-914,113	0	0.999	0	0	P2
19	SHALIMAR-2	0.001	0.998	0.001	0.001	P2
20	BLACK PLUM	0	0.999	0	0	P2
21	EC-528,360	0	0.999	0	0	P2
22	EC-528,372	0	1	0	0	P2
23	LOCAL 1	0	0.954	0	0.045	P2
24	POLISH TOMATO	0	0.999	0	0	P2
25	TOLCV-32	0.001	0.996	0.001	0.002	P2
26	EC-914,103	0	0.999	0	0	P2
27	IIHR-1939	0	0.999	0	0.001	P2
28	IIHR-2805	0	0.999	0	0	P2
29	IARI-7	0	0.999	0	0	P2
30	SUB ARCTIC PLENTY	0	0.999	0	0	P2
31	EC-164,334	0	0.999	0	0	P2
32	IARI-3	0.001	0.838	0.001	0.16	P2
33	STUPICE	0	0.998	0	0.002	P2
34	EC-914,106	0.001	0.999	0	0.001	P2
35	EC-521,078	0	0.999	0	0	P2
36	EC-914,108	0.001	0.998	0.001	0.001	P2
37	EC-914,092	0	0.999	0	0	P2
38	SHALIMAR-1	0.001	0.996	0.001	0.001	P2
39	WIR-13,706	0	0.999	0	0	P2
40	LOCAL 2	0	0.999	0	0	P2
41	MORTGAGE LIFTER	0	0.999	0	0	P2
42	EC-808,922	0	0.999	0	0.001	P2
43	S-22	0.001	0.997	0.001	0.001	P2
44	EC-914,091	0	0.999	0	0	P2
45	IARI-4	0.001	0.997	0.001	0.001	P2
46	WIR-13,708	0	0.999	0	0	P2
47	EC-914,112	0	0.999	0	0	P2
48	IHR-2201	0.001	0.998	0.001	0.001	P2
49	PUSA SHEETAL	0.001	0.998	0.001	0.001	P2
50	EC-145,057	0	1	0	0	P2

**Fig. 4:**
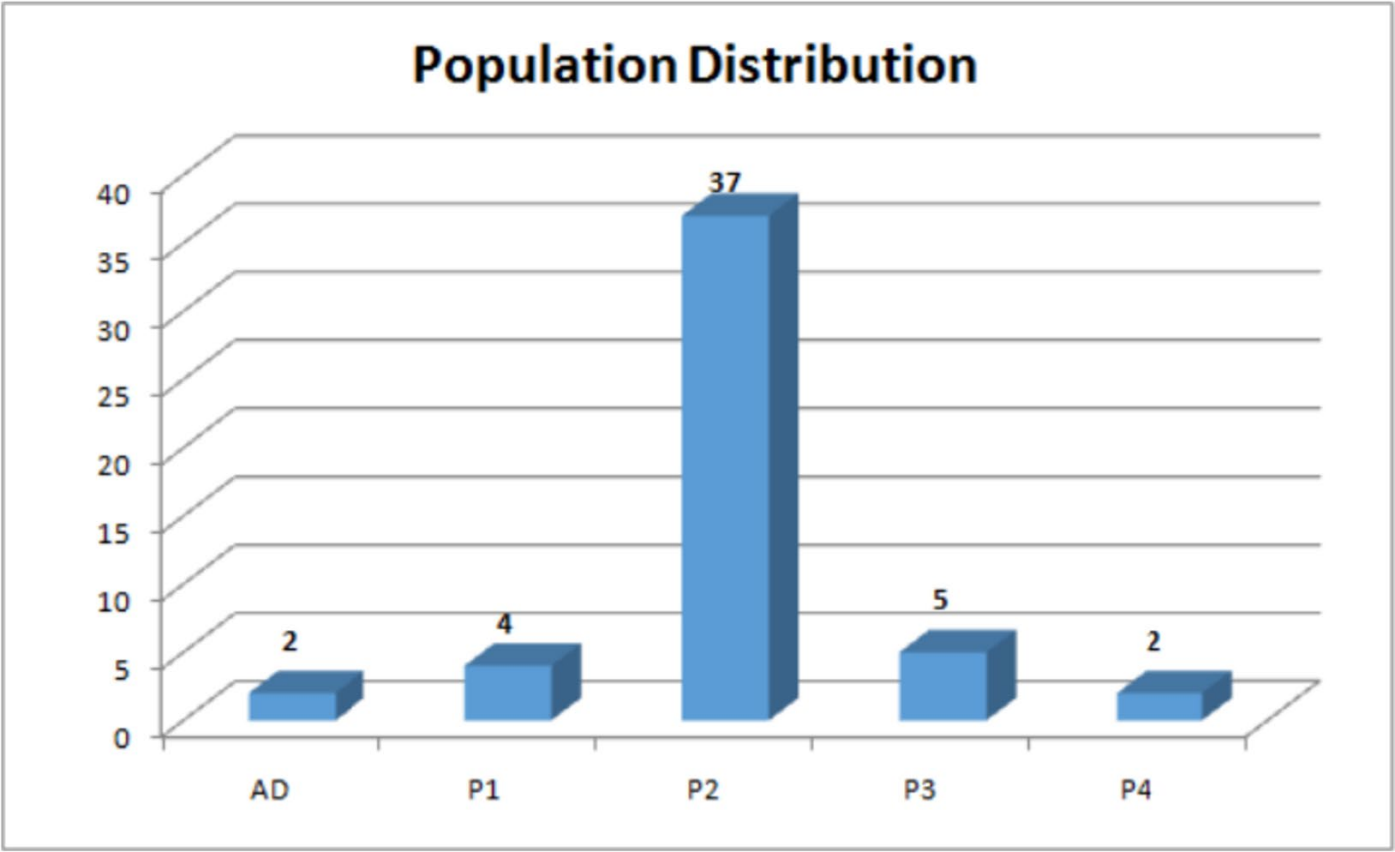
Number of genotypes in each subpopulation based on membership coefficient obtained using STRUCTURE software

These four subpopulations (Fig. 4) possessed an uneven distribution of genotypes with P2 receiving the maximum genotypes equal to 37. P3, P1 and P4 received 5, 4, 2 genotypes respectively and the population consisted of 2 admixtures.

**Fig. 5:**
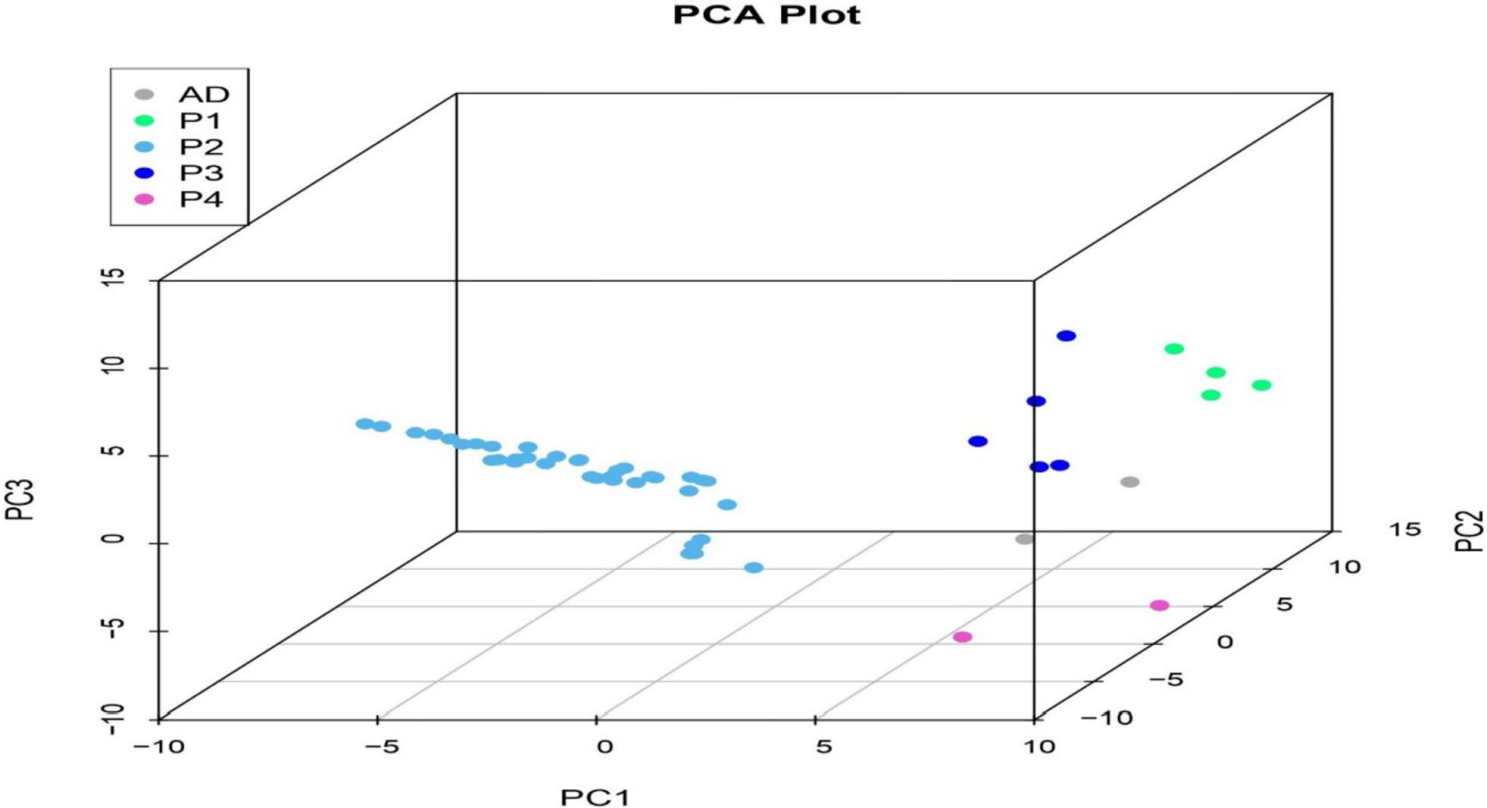
Principal component analysis using genotypic data from all the samples showed samples were scattered in different populations. Each dot represents an entry. Sub populations as defined by STRUCTURE analysis. D: admixed sub-population, P1: sub-population 1, P2: sub-population 2, P3: sub-population 3

**Fig. 6:**
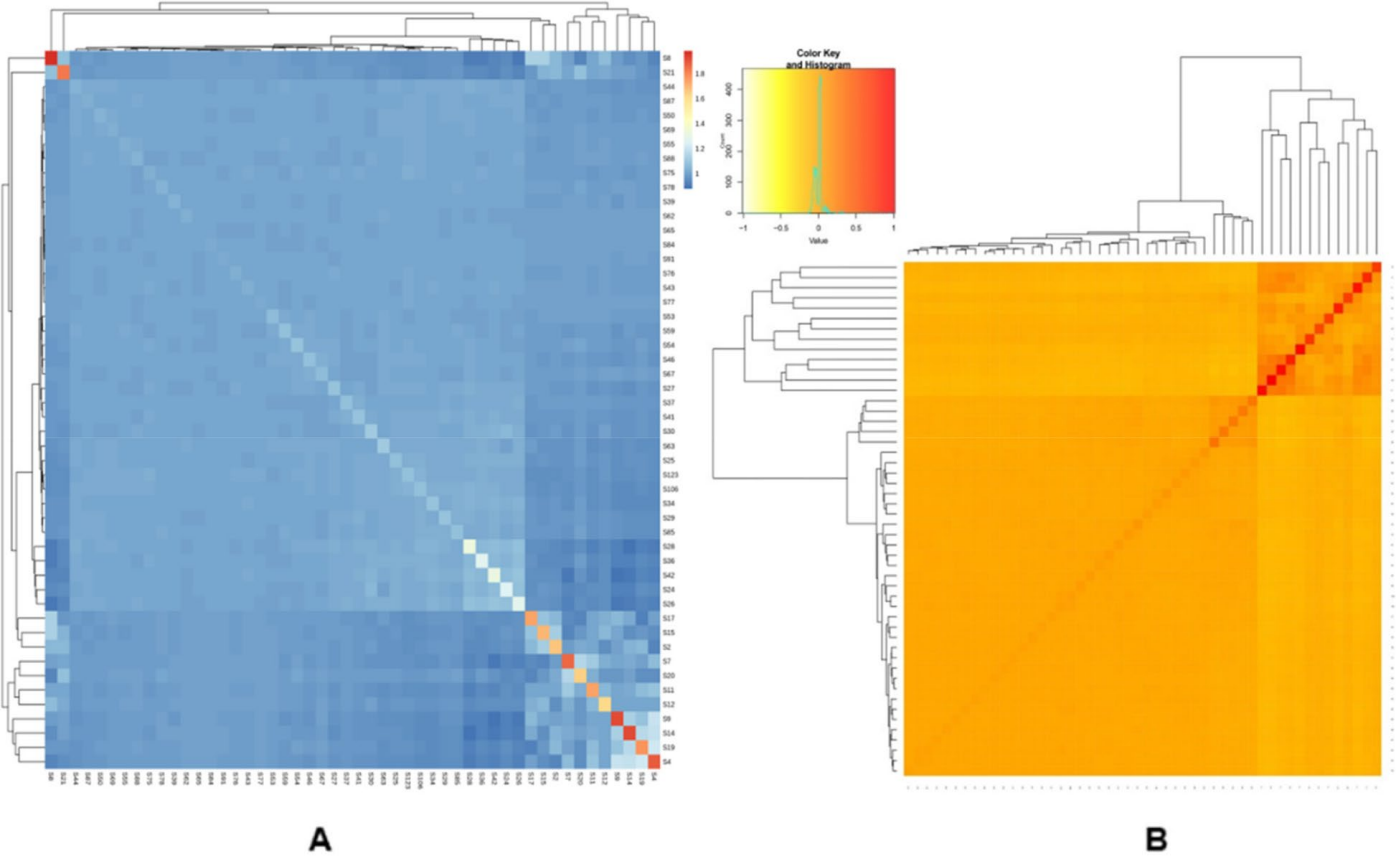
**A** Kinship plot generated from TASSEL, **B** Kinship plot generated from GAPIT

P2 consisted of majority of germplasm including exotic collections, land races, released varieties belonging majorly to species *Solanum lycopersicum* (31) and some of them to other species; *Solanum pimpinellifolium (2), Solanum. lycopersicum* var. *cerasiforme (3), Solanum peruvianum (1).* Other subpopulation that is P1 consisted of 4 genotypes belonging to species *Solanum peruvianum* (1), *Solanum habrochites* (1) and *Solanum lycopersicum* (2). P3 consisted of 5 genotypes belonging to *Solanum. lycopersicum* var. *cerasiforme* (2), *Solanum chilense* (1) and *Solanum lycopersicum* (2). P4 and admixtures both consisted of 2 genotypes both belonging to *Solanum lycopersicum*. There was no clear trend of the distribution. Through principal component analysis (Fig. 5) and Kinship analysis generated using trait analysis by association, evolution and linkage (TASSEL) (Fig. 6A) and genome association and prediction integrated tool (GAPIT) (Fig. 6B) analysis presence of four populations was confirmed.

### Genome wide association study

Then Tassel pipeline resulted in generation of 10,802 variants on aligning against the tomato reference genome. These variants were filtered to retain only SNPs which were to be further used for downstream analysis GWAS analysis. After filtration, 3854 SNPs were retained. SNPs were not distributed evenly across all chromosomes. The top significant marker trait associations are presented in Table 5.

**Table 5:** Top significant marker trait associations under different environments

S. No	ENV	Trait name	SNPs	Chromosome	Position	p-value	maf	Effect	PVE (%)
1	E3	Proline content	CH08_9484802	chr8	9,484,802	8.75*10^−6^	0.48	-0.73699	48.34
2	E3	Proline content	CH08_9484810	chr8	9,484,810	8.75*10^−6^	0.48	0.73699	48.34
3	E3	Proline content	CH08_9528421	chr8	9,528,421	7.65*10^−5^	0.44	-0.60225	36.37
4	E2	No of fruits per plant	CH12_49436823	chr12	49,436,823	4.18*10^−5^	0.5	204.3378	45.26
5	E4	No of fruits per plant	CH12_49436823	chr12	49,436,823	1.11*10^−4^	0.5	307.8472	38.19
6	E5	No of fruits per plant	CH12_49436823	chr12	49,436,823	8.19*10^−4^	0.5	151.2753	27.97
7	E5	No of days to first fruit set	CH09_32796951	chr9	32,796,951	2.4721*10^−4^	0.43	-9.63575	27.39
8	E5	No of days to first fruit set	CH11_13438935	chr11	13,438,935	7.27*10^−4^	0.38	-8.06909	20.52
9	E4	Fruit shape index	CH08_9435068	chr8	9,435,068	8.31*10^−7^	0.5	16.60184	26.22
10	E4	Fruit shape index	CH08_9452288	chr8	9,452,288	1.75*10^−5^	0.47	9.82668	18.52
11	E4	Fruit shape index	CH08_9452289	chr8	9,452,289	1.75*10^−5^	0.47	9.82668	18.52
12	E4	Fruit shape index	CH08_9454260	chr8	9,454,260	2.31*10^−5^	0.46	7.677636	17.88
13	E4	Fruit shape index	CH08_9454275	chr8	9,454,275	2.31*10^−5^	0.46	7.677636	17.88

E2: Polyhouse sown (2019); E3: Field, early sown (2020); E4: Field, normal sown (2020); E5: Field, late sown (2020)

### Marker trait association for cold tolerance traits

#### Pollen viability

A total of 265 significant SNPs were mapped for this trait under all the six environments. Under the cold stressed environments (E3 and E5) of open field conditions 119 significant SNPs were mapped. The mapping of significant SNPs was onto chromosomes 1, 2, 8, 9, 11 and 12 mostly. CH09_32765160, CH09_32765161 and CH09_32765231 were mapped across both the protected environments (E2 and E6) in both years explaining major variation of > 10% with additive effect. CH08_9512860 was mapped across both the normal environments (E1 and E4) in both years explaining a major phenotypic variation of > 10% (PVE) with negative effect. CH08_9396204 was mapped across both the cold stressed environments (E3 and E5) with explaining a major phenotypic variation of > 10% with additive effect.

#### Malondialdehyde

A total of 728 significant SNPS were mapped for this trait under all the six environments. Under the cold stressed environments (E3 and E5) of open field conditions 238 significant SNPs were mapped. The mapping of significant SNPs was mostly onto chromosomes 1, 6, 2, 8, 9 and 11. CH08_9395919 and CH02_22999374, were mapped across both the normal environments (E1 and E4) in both years explaining a major phenotypic variation of > 10% (PVE) with negative effect which is desirable for the stated trait. CH08_9513555, CH08_9513565 and CH02_22989441 were mapped across both the protected environments (E2 and E6) in both years explaining major variation of > 10% with negative effect. CH08_9292940 was mapped across both the cold stressed environments with additive variance explaining a minor phenotypic variation of > 10% with negative effect.

**Figure:**
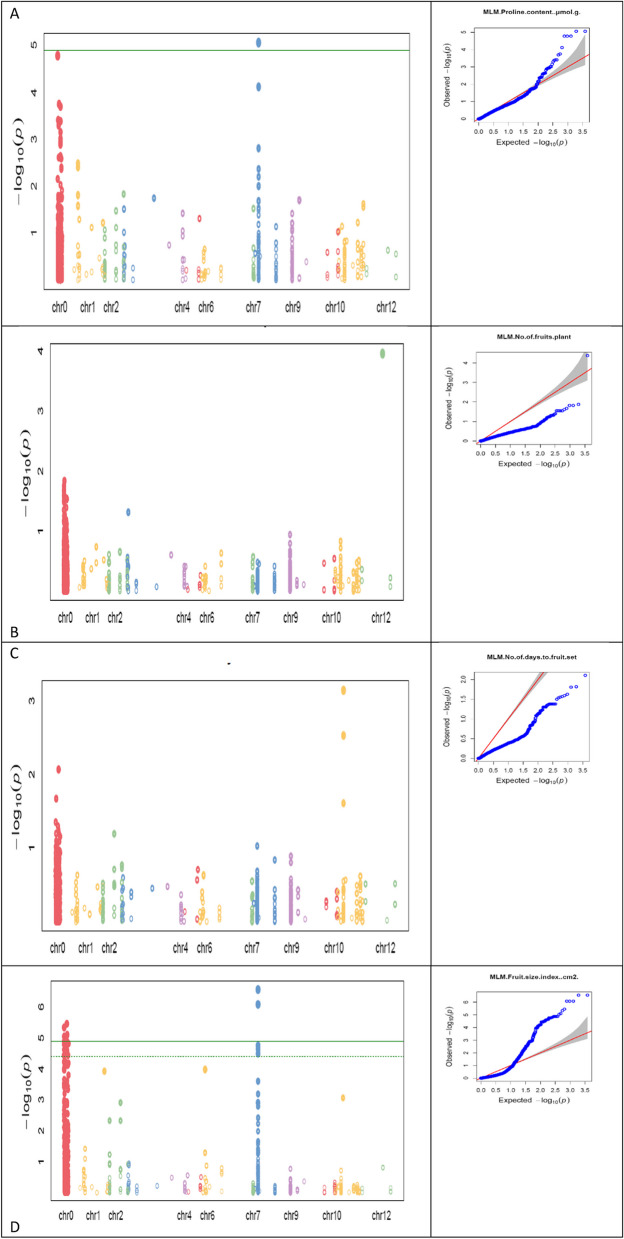
Manhattan plots and Quantile-Quantile (QQ) plots of top significant markers for **A** Proline content, **B** No of fruits per plant, **C** No of days to first fruit set, **D** Fruit shape index under different environments

#### Proline content

A total of 757 significant SNPs were mapped for this trait under all the six environments. Under the cold stressed environments (E3 and E5) of open field conditions 275 significant SNPS were mapped. The mapping of significant SNPs was onto chromosome 8 and 11 mostly. CH08_9484802 and CH08_9484810 were mapped across both the cold stressed environments (E3 and E5) with explaining a major phenotypic variation of > 10% with the former having negative effect and latter having additive effect. CH08_9484802 (8.75*10 − 6), CH08_9484810 (8.75*10 − 6) and CH08_9528421 (7.65*10 − 5) were mapped across early cold environment with highly significant P-values. CH08_9456588 and CH08_9435068 were mapped across both the protected environments (E2 and E6) in both years explaining major variation of > 10% with additive effect. CH08_9435068 was mapped across both the normal environments (E1 and E4) in both years explaining a major phenotypic variation of > 10% (PVE) with additive effect.

#### Total leaf chlorophyll content

A total of 163 significant SNPs were mapped for this trait under all the six environments. Under the cold stressed environments (E3 and E5) of open field conditions 35 significant SNPs were mapped. The mapping of significant SNPS for total leaf chlorophyll content was onto chromosomes 1, 2, 3, 8, 9, 10 and 11 mostly. CH08_9408610, CH09_32797168, CH09_32797221 and CH09_32797222 were mapped across both the normal environments (E1 and E4) in both years explaining a minor phenotypic variation of < 10% (PVE) with additive effect. CH09_32800680, CH09_32800681 and CH09_32797168 were mapped across both the protected environments (E2 and E6) in both years explaining major variation of > 10% with additive effect.

#### Ascorbic acid

A total of 792 significant SNPS were mapped for this trait under all the six environments. Under the cold stressed environments (E3 and E5) of open field conditions 236 significant SNPS were mapped. The mapping of significant SNPs was mostly onto chromosomes 2, 3, 9, 10 and 11. Some of the top significant SNPs across environments overlapped, indicating some major effect loci. CH11_51779471 was mapped across all the six environments explaining a major phenotypic variation of > 10% (PVE) with additive effect. CH10_64514613 and CH10_64514614 were mapped across both the normal environments (E1 and E4) in both years explaining minor phenotypic variation of < 10% with additive effects. CH02_4489 and CH09_32801508 were mapped across both the protected environments (E2 and E6) in both years explaining major variation of > 10%. CH11_51768254 was mapped across both the cold stressed environments with additive variance explaining a minor phenotypic variation of < 10%.

#### Lycopene content

A total of 799 significant SNPs were mapped for this trait under all the six environments. Under the cold stressed environments (E3 and E5) of open field conditions 314 significant SNPS were mapped. The mapping of significant SNPs was onto chromosomes 2, 5, 6, 9 and 11 mostly. CH06_10446131 and CH06_10446132 were mapped across five environments except the late sown environment explaining a major phenotypic variation of > 10% (PVE) and additive effect. CH11_13438931 was mapped across both the normal environments (E1 and E4) in both years explaining a major phenotypic variation of > 10% (PVE) with additive effect. CH11_2043421 was mapped across both the protected environments (E2 and E6) in both years explaining major variation of > 10% with additive effect.

#### Total phenols

A total of 643 significant SNPs were mapped for this trait under all the six environments. Under the cold stressed environments (E3 and E5) of open field conditions 263 significant SNPs were mapped. The mapping of significant SNPs was onto chromosome 2, 3, 6, 7, 8, 9 and 11 mostly. CH06_8656494, CH06_8656496 and CH07_67281761 were mapped across both the normal environments (E1 and E4) in both years explaining a minor phenotypic variation of < 10% (PVE) with additive effect. CH03_542789 and CH09_32765930 were mapped across both the protected environments (E2 and E6) in both years explaining major variation of > 10% with additive effect.

#### Total soluble sugars

A total of 370 significant SNPs were mapped for this trait under all the six environments. Under the cold stressed environments (E3 and E5) of open field conditions 206 significant SNPS were mapped. The mapping of significant SNPs was onto chromosomes 2, 6, 8, 9 and 11 mostly. CH08_9452288, CH08_9452289 and CH06_43801072 were mapped across both the normal environments (E1 and E4) in both years explaining a minor phenotypic variation of < 10% (PVE) with additive effect. CH02_22990072 and CH11_13438935 were mapped across both the protected environments (E2 and E6) in both years explaining minor phenotypic variation of < 10% with additive effect.

### Marker trait association for yield and yield related traits

#### Days to emergence

A total of 456 significant SNPs were mapped for this trait under all the six environments. Under the cold stressed environments (E3 and E5) of open field conditions 85 significant SNPS were mapped. The mapping of significant SNPs was onto chromosomes 1, 2, 6, 7, 8 9 and 11 mostly. CH09_32800889 and CH09_32800890 were mapped across both the normal environments (E1 and E4) in both years explaining a minor phenotypic variation of < 10% (PVE) with negative effect desirable for the trait. CH06_11575381, CH06_11575394, CH06_11575453 and CH06_8639002 were mapped across both the protected environments (E2 and E6) in both years explaining minor phenotypic variation of < 10% with negative effect.

#### Seedling length at transplant

A total of 702 significant SNPs were mapped for this trait under all the six environments. Under the cold stressed environments (E3 and E5) of open field conditions 309 significant SNPS were mapped. The mapping of significant SNPs for this trait was onto chromosomes 4, 5, 6, 8, 9 and 11 mostly. CH05_26702700, CH05_26702702 and CH08_9396204 were mapped across both the normal environments (E1 and E4) in both years explaining a major phenotypic variation of > 10% (PVE) with additive effect.

#### Number of flowers per truss

A total of 187 significant SNPs were mapped for this trait under all the six environments. Under the cold stressed environments (E3 and E5) of open field conditions 71 significant SNPS were mapped. The mapping of significant SNPs was onto chromosome 3, 8, 9, 11 and 12 mostly. CH03_2152187, CH08_9512856, CH09_32801176, CH09_32801177 and CH09_32801178 was mapped across both the protected environments (E2 and E6) in both years explaining major phenotypic variation of > 10% with additive effect.

#### Number of days to first fruit set

A total of 365 significant SNPs were mapped for this trait under all the six environments. Under the cold stressed environments (E3 and E5) of open field conditions 25 significant SNPs were mapped. The mapping of significant SNPS was onto chromosomes 2, 6, 8, 9, 11 and 12 mostly. CH09_32796951, CH09 32,796,992 and CH09_32797021 were mapped across both the normal environments (E1 and E4) in both years explaining a major phenotypic variation of > 10% (PVE) with negative effect desirable for the trait.

#### Number of fruits per truss

A total of 362 significant SNPs were mapped for this trait under all the six environments. Under the cold stressed environments (E3 and E5) of open field conditions 31 significant SNPs were mapped. The mapping of significant SNPS was onto chromosome 1, 3, 7, 9, 11 and 12 mostly. CH12_49436823 was mapped across both the protected environments (E2 and E6) in both years explaining minor phenotypic variation of < 10% with additive effect.

#### Number of days to first harvest

A total of 265 significant SNPs were mapped for this trait under all the six environments. Under the cold stressed environments (E3 and E5) of open field conditions 35 significant SNPS were mapped. The mapping of significant SNPs was onto chromosomes 1, 2, 3, 8, 9 and 11 mostly. CH11_13438926 was mapped across both the protected environments (E2 and E6) in both years explaining major phenotypic variation of > 10%.

#### Average fruit weight

A total of 1085 significant SNPs were mapped for this trait under all the six environments. Under the cold stressed environments (E3 and E5) of open field conditions 346 significant SNPS were mapped. The mapping of significant SNPs was onto chromosomes 7, 8, 9 and 11 mostly. CH09_32800889, CH09_32800890, CH11_51768254 and CH11_51768333 were mapped across all the environments except the late sown environment in both years explaining minor phenotypic variation of < 10% with negative effect.

#### Fruit shape index

A total of 793 significant SNPS were mapped for this trait under all the six environments. Under the cold stressed environments (E3 and E5) of open field conditions 211 significant SNPS were mapped. The mapping of significant SNPs was onto chromosomes 1, 2, 3, 4, 6, 8, 9 and 11 mostly. Different SNPs were mapped across different environments. Top SNPS with highly significant p-values were mapped across the normal environment (E4) namely CH08_9435068 (8.31*10 − ^7^), CH08_9452288 (1.75*10 − ^5^), CH08_9452289 (1.75*10 − ^5^), CH08_9454260 (2.31*10 − ^5^) and CH08_9454275 (2.31*10 − ^5^) explaining major phenotypic variation of > 10% with additive effect.

#### Number of fruits per plant

A total of 289 significant SNPs were mapped for this trait under all the six environments. Under the cold stressed environments (E3 and E5) of open field conditions 158 significant SNPS were mapped. The mapping of significant SNPs was onto chromosomes 2, 3, 9, 10, 11 and 12 mostly. CH03_2152187 was mapped across both the normal environments (E1 and E4) in both years explaining a major phenotypic variation of > 10% (PVE) with additive effect. CH12_49436823 was mapped across both the cold environments (E3 and E5) in both years explaining a major phenotypic variation of > 10% (PVE) with additive effect.

#### Fruit yield per plant

A total of 701 significant SNPs were mapped for this trait under all the six environments. Under the cold stressed environments (E3 and E5) of open field conditions 101 significant SNPS were mapped. The mapping of significant SNPs was on to chromosomes 1, 4, 7, 8, 9 and 11 mostly. CH04_58505565 was mapped across both the normal environments (E1 and E4) in both years explaining a major phenotypic variation of > 10% (PVE) with additive effect. CH04_58505565 and CH01_60016947 were mapped across both the protected environments (E2 and E6) in both years explaining major phenotypic variation of > 10% with additive effect.

#### Number of primary branches

A total of 469 significant SNPs were mapped for this trait under all the six environments. Under the cold stressed environments (E3 and E5) of open field conditions 114 significant SNPS were mapped. The mapping of significant SNPs was onto chromosome 1, 2, 8, 9 and 11 mostly. CH08_9792846 was mapped across both the protected environments (E2 and E6) in both years explaining major phenotypic variation of > 10% with additive effect.

#### Plant height

A total of 610 significant SNPs were mapped for this trait under all the six environments. Under the cold stressed environments (E3 and E5) of open field conditions 210 significant SNPS were mapped. The mapping of significant SNPs was onto chromosomes 1, 2, 6, 8 and 9 mostly. CH08_9792846 was mapped across both the protected environments (E2 and E6) in both years explaining a major phenotypic variation of > 10% (PVE) with additive effect. CH06_43801076 was mapped across both the cold environments (E3 and E5) in both years explaining a major phenotypic variation of > 10% (PVE) with additive effect.

#### Duration of harvest

A total of 145 significant SNPs were mapped for this trait under all the six environments. Under the cold stressed environments (E3 and E5) of open field conditions 85 significant SNPS were mapped. The mapping of significant SNPs was onto chromosomes 1, 2, 8, 9 and 11 mostly. CH02_11280 and CH08_9792846 were mapped across both the normal environments (E1 and E4) in both years explaining a minor phenotypic variation of < 10% (PVE) with additive effect. CH11_13438944, was mapped across both the protected environments (E2 and E6) in both years explaining minor phenotypic variation of < 10% with additive effect.

### Candidate genes

Mining of the candidate genes revealed a total of 685 genes; 40 genes from physical positions 24,506,038–86,800,901 bp on chromosome 1, 72 genes from physical positions 2344-38613228 bp on chromosome 2, 22 genes from physical positions 542789-61706899 bp on chromosome 3, 13 genes from physical positions 30,904,930–64,421,176 bp on chromosome 4, 8 genes from physical positions 1,507,699–28,259,872 bp on chromosome 5, 36 genes from physical positions 4,370,518–43,806,292 bp on chromosome 6, 38 genes from physical positions 66,235,127–67,281,761 bp on chromosome 7, 190 genes from physical positions 2,628,180–45,067,799 bp on chromosome 8, 137 genes from physical positions 23,454,319–61,706,964 bp on chromosome 9, 16 genes from physical positions 42,879,485–64,514,640 bp on chromosome 10, 108 genes from physical positions 2,043,342–52,369,820 bp on chromosome 11 and 7 genes from physical positions 5,896,180–65,817,136 bp on chromosome 12. Based on functional categorization, 60 genes were found to be associated with biological processes in these genomic regions (5 on chromosome-1, 8 on chromosome-2, 1 on chromosome-3, 4 on chromosome-4, 2 on chromosome-5, 3 on chromosome-6, 2 on chromosome-7, 15 on chromosome-8, 6 on chromosome-9, 2 on chromosome-10, 10 on chromosome-11 and 2 on chromosome-12). 7 among 60 were directly found to be related to abiotic stress tolerance and function directly or indirectly as cold stress responsive genes. First among seven genes comprised of Solyc03g007550.4 annotated LIM domain protein (AHRD V3.3 *** AT5G52950.1). Second one was Solyc11g039880.2 with annotation as Nucleoporin (DUF3414) (AHRD V3.3 *** F4JUG3_ARATH). Third one was Solyc02g022930.3 with annotation as 3-hydroxyisobutyrate dehydrogenase (AHRD V3.3 *** A0A1U8FBY0_CAPAN) and fourth one was Solyc07g065840.2 annotated as molecular chaperone Hsp90-2. Fifth one was Solyc04g082980.2 annotated as Tetratricopeptide repeat protein SKI3 (AHRD V3.3 *** A0A2G2ZSZ3_CAPAN) and sixth one was Solyc05g006830.3 annotated as Thioredoxin (AHRD V3.3 *** A0A2G2VM05_CAPBA). Seventh one was Solyc11g017345.1 annotated as F-box/FBD/LRR-repeat protein (AHRD V3.3 *-* XP_010312830.1). Other genes included essential genes for growth and developmental processes (Table 6).

**Table 6:** List of candidate genes identified

S.No	Trait	SNP	Chromosome	Candidate gene	Candidate Gene Annotation
1	Germination percentage, Days to emergence, Number of fruits per truss, Number of fruits per plant, Total leaf chlorophyll content	CH09_32797168	9	Solyc09g050070.1 (ITAG4.0)	NAD(P)H-quinone oxidoreductase subunit 2, chloroplastic (AHRD V3.3 *** A0A2P1N7K1_9LAMI)
2	Germination percentage, Seedling vigor index, Duration of harvest	CH08_9528498	8	Solyc08g016796.1 (ITAG4.0)	UNKNOWN PROTEIN
3	Germination percentage	CH08_9481068	8	Solyc08g150109.1 (ITAG4.0)	UNKNOWN PROTEIN
4	Seedling vigor index, No of fruits per truss, Duration of harvest,	CH11_51779471	11	Solyc11g150146.1 (ITAG4.0)	Elongation factor 1-alpha (AHRD V3.3 *** A0A1U8EL12_CAPAN)
5	Seedling vigor index, Ascorbic acid, Lycopene content	CH11_2043421	11	Solyc11g007770.2 (ITAG4.0)	Glycosyltransferase family protein 64 protein C5 (AHRD V3.3 *** A0A2G2VEI7_CAPBA)
6	Seedling vigor index, Duration of harvest	CH08_45067778	8	Solyc08g061145.1 (ITAG4.0)	Geraniol 8-hydroxylase (AHRD V3.3 *-* A0A2G2W2C6_CAPBA)
7	Seedling vigor index	CH01_34883400	1	Solyc01g038232.1 (ITAG4.0)	UNKNOWN PROTEIN
8	Days to emergence, Average fruit weight	CH09_32800889	9	Solyc09g050070.1 (ITAG4.0)	NAD(P)H-quinone oxidoreductase subunit 2, chloroplastic (AHRD V3.3 *** A0A2P1N7K1_9LAMI)
9	Days to emergence	CH07_66235223	7	Solyc07g064130.2 (ITAG4.0)	Polyubiquitin (AHRD V3.3 *** F1SY21_MESCR)
10	Days to emergence	CH08_45067768	8	Solyc08g061145.1 (ITAG4.0)	Geraniol 8-hydroxylase (AHRD V3.3 *-* A0A2G2W2C6_CAPBA)
11	Days to emergence, Seedling length at transplant	CH06_11575381	6	Solyc06g016760.3 (ITAG4.0)	Gamma-secretase subunit (AHRD V3.3 *-* AT5G09310.1)
12	Days to emergence	CH02_12004	2	Solyc02g004000.1 (ITAG4.0)	Regulator of rDNA transcription protein 15 (AHRD V3.3 *-* A0A2G2V0S9_CAPBA)
13	Days to emergence	CH06_8639002	6	Solyc06g150106.1 (ITAG4.0)	UNKNOWN PROTEIN
14	Days to emergence	CH08_9395894	8	Solyc08g016791.1 (ITAG4.0)	UNKNOWN PROTEIN
15	Days to emergence, Fruit yield per plant	CH08_9516336	8	Solyc08g016796.1 (ITAG4.0)	UNKNOWN PROTEIN
16	Days to emergence, Number of days to first harvest, Average fruit weight, Ascorbic acid.	CH11_51768254	11	Solyc11g150146.1 (ITAG4.0)	Elongation factor 1-alpha (AHRD V3.3 *** A0A1U8EL12_CAPAN)
17	Seedling length at transplant	CH05_26702700	5	Solyc05g021190.2 (ITAG4.0)	Photosystem II D2 protein (AHRD V3.3 *-* A0A2G2XVH6_CAPAN)
18	Seedling length at transplant, Fruit shape index, Total leaf chlorophyll content	CH11_8317802	11	Solyc11g017345.1 (ITAG4.0)	F-box/FBD/LRR-repeat protein (AHRD V3.3 *-* XP_010312830.1)
19	Seedling length at transplant, Fruit yield per plant	CH04_58505565	4	Solyc04g074580.1 (ITAG4.0)	Histone H3 (AHRD V3.3 *** A0A2K1J132_PHYPA)
20	Seedling length at transplant, Total soluble sugars	CH09_32793167	9	Solyc09g050069.1 (ITAG4.0)	NAD(P)H-quinone oxidoreductase subunit 2, chloroplastic (AHRD V3.3 *-* A0A0U2L2P8_9MYRT)
21	Seedling length at transplant, Number of primary branches	CH04_30904930	4	Solyc04g024420.3 (ITAG4.0)	Proteasome subunit beta (AHRD V3.3 *** A0A2G2VMP0_CAPBA)
22	Seedling length at transplant	CH02_38534615	2	Solyc02g071000.1 (ITAG4.0)	Chlorophyll a-b binding protein, chloroplastic (AHRD V3.3 *** A0A2G3A7J8_CAPAN)
23	Seedling length at transplant, Plant height, Duration of harvest	CH02_11280	2	Solyc02g004000.1 (ITAG4.0)	Regulator of rDNA transcription protein 15 (AHRD V3.3 *-* A0A2G2V0S9_CAPBA)
24	Seedling length at transplant, Number of days to first fruit set, Total soluble sugars	CH11_47595957	11	Solyc11g063480.1 (ITAG4.0)	UNKNOWN PROTEIN
25	Number of flowers per truss, Number of fruits per truss, Number of fruits per plant, Fruit yield per plant, Ascorbic acid	CH03_2152187	3	Solyc03g007550.4 (ITAG4.0)	LIM domain protein (AHRD V3.3 *** AT5G52950.1)
26	Number of flowers per truss, Number of fruits per truss	CH08_9512856	8	Solyc08g016796.1 (ITAG4.0)	UNKNOWN PROTEIN
27	Number of flowers per truss	CH11_40366610	11	Solyc11g039880.2 (ITAG4.0)	Nucleoporin (DUF3414) (AHRD V3.3 *** F4JUG3_ARATH)
28	Number of flowers per truss, Number of days to first fruit set, Number of fruits per truss, Number of fruits per plant, Pollen viability and Ascorbic acid	CH12_49436823	12	Solyc12g038400.2 (ITAG4.0)	Acetyl-coenzyme A synthetase (AHRD V3.3 *** A4W5I5_ENT38)
29	Number of days to first fruit set	CH06_10446133	6	Solyc06g024320.1 (ITAG4.0)	UNKNOWN PROTEIN
30	Number of days to first fruit set, Number of days to first harvest	CH08_9570356	8	Solyc08g150111.1 (ITAG4.0)	UNKNOWN PROTEIN
31	Number of days to first fruit set, Malondialdehyde	CH11_13443107	11	Solyc11g021180.2 (ITAG4.0)	Protein TIC 214 (AHRD V3.3 *-* J7H3X3_CAPAN)
32	Number of days to first fruit set, Pollen viability, Malondialdehyde	CH02_22989441	2	Solyc02g022930.3 (ITAG4.0)	3-hydroxyisobutyrate dehydrogenase (AHRD V3.3 *** A0A1U8FBY0_CAPAN)
33	Number of fruits per truss, Fruit shape index and Number of primary branches	CH01_60016916	1	Solyc01g058470.1 (ITAG4.0)	UNKNOWN PROTEIN
34	Number of fruits per truss, Average fruit weight, Total phenols.	CH09_32765930	9	Solyc09g050061.1 (ITAG4.0)	UNKNOWN PROTEIN
35	Number of days to first harvest, Total soluble sugars	CH11_47631045	11	Solyc11g063577.1 (ITAG4.0)	Protein Ycf2 (AHRD V3.3 *-* A0A142DP97_9LAMI)
36	Number of days to first harvest	CH02_38613228	2	Solyc02g071110.3 (ITAG4.0)	Purine permease (AHRD V3.3 *** A0A2R6Q6Y9_ACTCH),Pfam:PF16913
37	Number of days to first harvest, Proline content	CH08_9395918	8	Solyc08g016791.1 (ITAG4.0)	UNKNOWN PROTEIN
38	Number of days to first harvest	CH08_9502536	8	Solyc08g016794.1 (ITAG4.0)	UNKNOWN PROTEIN
39	Number of days to first harvest	CH09_47357531	9	Solyc09g057860.1 (ITAG4.0)	UNKNOWN PROTEIN
40	Average fruit weight, Fruit yield per plant	CH07_67281689	7	Solyc07g065840.2 (ITAG4.0)	Molecular chaperone Hsp90-2
41	Average fruit weight	CH08_9514308	8	Solyc08g016796.1 (ITAG4.0)	UNKNOWN PROTEIN
42	Fruit shape index	CH04_61558849	4	Solyc04g078900.3 (ITAG4.0)	ABA 8'-hydroxylase
43	Fruit shape index	CH01_81577621	1	Solyc01g098950.3 (ITAG4.0)	Glyceraldehyde-3-phosphate dehydrogenase (AHRD V3.3 *** A0A2G3C9S8_CAPCH)
44	Fruit shape index, Number of primary branches, Plant height, Duration of harvest.	CH08_9792844	8	Solyc08g016806.1 (ITAG4.0)	UNKNOWN PROTEIN
45	Fruit shape index	CH09_47357555	9	Solyc09g057860.1 (ITAG4.0)	UNKNOWN PROTEIN
46	Number of fruits per plant, Ascorbic acid	CH10_64514613	10	Solyc10g086580.2 (ITAG4.0)	Ribulose bisphosphate carboxylase/oxygenase activase, chloroplastic (AHRD V3.3 *** RCA_SOLPN)
47	Fruit yield per plant	CH01_83040089	1	Solyc01g100860.3 (ITAG4.0)	ADP-ribosylation factor (AHRD V3.3 *** B4FP40_MAIZE)
48	Fruit yield per plant, Plant height	CH12_5896264	12	Solyc12g015880.2 (ITAG4.0)	Heat shock protein 90-1
49	Fruit yield per plant	CH11_47615653	11	Solyc11g063573.1 (ITAG4.0)	ATPase subunit 4 (AHRD V3.3 *** A0A0C5B288_HYONI)
50	Fruit yield per plant	CH08_9292962	8	Solyc08g016780.1 (ITAG4.0)	UNKNOWN PROTEIN
51	No of primary branches, Duration of harvest	CH02_23061139	2	Solyc02g023940.3 (ITAG4.0)	UNKNOWN PROTEIN
52	Pollen viability	CH04_64421176	4	Solyc04g082980.2 (ITAG4.0)	Tetratricopeptide repeat protein SKI3 (AHRD V3.3 *** A0A2G2ZSZ3_CAPAN)
53	Malondialdehyde	CH02_23029093	2	Solyc02g022935.1 (ITAG4.0)	Golgi-associated plant pathogenesis-related protein 1 (AHRD V3.3 --* A0A226DTU7_FOLCA)
54	Proline content	CH08_9435066	8	Solyc08g016792.1 (ITAG4.0)	UNKNOWN PROTEIN
55	Proline content, Total leaf chlorophyll content.	CH08_9484802	8	Solyc08g150109.1 (ITAG4.0)	UNKNOWN PROTEIN
56	Proline content	CH01_32207691	1	Solyc01g021620.3 (ITAG4.0)	Dirigent protein (AHRD V3.3 *** H8ZXA8_SOLTU)
57	Total leaf chlorophyll content, total phenols	CH10_42879486	10	Solyc10g048060.2 (ITAG4.0)	Hypothetical protein (AHRD V3.3 *-* ATMG00030.1)
58	Lycopene content	CH05_1507699	5	Solyc05g006830.3 (ITAG4.0)	Thioredoxin (AHRD V3.3 *** A0A2G2VM05_CAPBA)
59	Total phenols	CH02_23020130	2	Solyc02g022935.1 (ITAG4.0)	Golgi-associated plant pathogenesis-related protein 1 (AHRD V3.3 --* A0A226DTU7_FOLCA)
60	Total soluble sugars	CH11_17659987	11	Solyc11g051171.1 (ITAG4.0)	UNKNOWN PROTEIN

## Discussion

Every degree increase in ambient temperature, in the context of climate change, has a significant impact on crop yield, particularly for tomato, which is exceptionally sensitive to cold temperatures. Tomato cultivar’s low temperature sensitivity limits their geographic distribution, cultivation and growing season. Therefore, understanding the nature, impact, and molecular mechanisms of cold stress tolerance will help in designing strategies to overcome production losses. Previously, no studies have been reported on GWAS for cold tolerance in tomato. Studies were more focused on understanding the nature, impact, and existing diversity in germplasm lines as well as identifying the genomic regions responsible for yield and quality traits. In this study, we reported some marker trait associations for cold stress tolerance in tomato.

The analysis of variance revealed highly significant differences among all the genotypes under study for all traits and environments thereby indicating a good amount of variability in the present material. The mean performance of genotypes for various traits under different environments showed large variation and also the range was high for almost all the traits under study indicating that wide variation existed in the population. Heritability in broad sense was found to be high for all the characters under study. High heritability for physiological traits indicated that the selection for cold tolerance relying on these traits could be effective. Its value ranged from 89 − 100%. Studies for varaiblity of germplasm were conducted by various workers viz., [23, 24] and were of the same opinion that there was presence of sufficient variability in the tomato germplasm.

Based on STRUCTURE, SNPhylo and Kinship matrix, the tomato (50 lines) germplasm belonging to different species was categorized into four distinct populations. These four subpopulations possessed an uneven distribution of genotypes with P2 receiving the maximum genotypes equal to 37. P3, P1 and P4 received 5, 4, 2 genotypes respectively and the population consisted of 2 admixtures. P2 consisted of majority of germplasm including exotic collections, land races, released varieties belonging majorly to species *Solanum lycopersicum* (31) and some of them to other species; *Solanum pimpinellifolium* (2), *Solanum lycopersicum var. cerasiforme* (3), *Solanum peruvianum* (1). The grouping of *Solanum pimpinellifolium*, in the same cluster with other *Solanum lycopersicum* lines was also reported by [25] and [26] as sister groups. Other subpopulation that is P1 consisted of 4 genotypes belonging to species *Solanum peruvianum* (1), a derivative of *Solanum habrochites* (1) and *Solanum lycopersicum* (2). The clustering of *Solanum chilense* and *Solanum habrochaites* together can possibly be due to their origin Southern Peru where they are mainly found [27]. P3 consisted of 5 genotypes belonging to *Solanum lycopersicum var. cerasiforme* (2), *Solanum chilense* (1) and *Solanum lycopersicum* (2). The grouping of *Solanum chilense* and *Solanum lycopersicum var. cerasiforme* together can be attributed to the complementary properties they posses for salt-stress resistance and smaller fruit size as proved by [28]. P4 consisted of 2 genotypes both belonging to *Solanum lycopersicum*. Admixtures consisted of 2 genotypes both of them being exotic collections belonging to species *Solanum lycopersicum*. The admixture level of first admixture obtained was higher in sub-population 1 and that of second admixture obtained was higher in sub-population 3 among 4 populations generated. The tendency of genotypes being distributed into various clusters by species indicated that the species were genetically unique. Clustering by origin region found that accessions with similar genetic similarity are also geographically similar. The distribution, on the other hand, lacked a distinct pattern. The genetic structure discovered in our study implies that selection for market specialisation has left a genetic trace in grown tomatoes. The observed variance also suggests that various patterns of genetic variation in farmed tomatoes are caused by wild species introgression and market specialised selection. Tomatoes (*Solanum lycopersicum*) have been subjected to extensive selection both during and after domestication.

Tomato has been a forerunner in QTL mapping for agronomic and yield-related traits on populations. Several genome wide association mapping investigations have been conducted in the previous decade, resulting in the discovery of novel loci for fruit quality, metabolites and flavor-related compounds. Efforts have also been made to explore major agronomic features in core sets of improved and wild cultivars. However, all the research was focused on yield and yield-related variables under normal conditions, resulting in the identification of several associated genomic areas, but no attempts were made to map loci under cold stress and protected environments. But this study, allowed us to identify marker trait associations for cold stress in tomato, since it was the first and one of its kind. In addition to reporting marker trait associations for cold tolerance traits, associations for yield and yield related traits under normal and cold stressed environments was also done which to the best of our knowledge, is the first comprehensive study.

Cold stress impairs the plant system and therefore there are substances which act as markers for susceptibility or resistance. Pollen viability, total leaf chlorophyll content and lycopene act as such markers and are all reduced in response to cold stress in susceptible plants as a response to cold stress. Poor winter fruit set in tomato has been reported by drop in both pollen quality and quantity [29] where in cold stress alters anther’s metabolic pathways, causing pollen sterility. Sharma KD. et al. [30] reported that unlike vulnerable plants, cold-tolerant plants produce a large amount of viable pollen. The mapping of significant SNPs was onto chromosomes 1, 2, 4, 8, 9, 11 and 12 mostly for pollen viability. Xu J. et al. [31] also mapped a single QTL for pollen viability on chromosome 11 in tomato. The gene Solyc04g082980.2 annotated as Tetratricopeptide repeat protein SKI3 was identified for the trait. Rosado A. et al [32] reported that Tetratricopeptide repeat protein regulates the transcript levels of several dehydration-responsive genes, such as the transcription factor *DREB2A* and genes encoding dehydration response proteins, such as *ERD1* (early response to dehydration 1), *ERD3* and *COR15a*. Furthermore Solyc02g022930.3 annotated as 3-hydroxyisobutyrate dehydrogenase was identified and was also colocalized for the trait malondialdehyde which is a marker of oxidative lipid injury. Schertl P. et al. [33] reported that under abiotic stress conditions in Arabidopsis, 3-hydroxyisobutyrate dehydrogenase acts as a NADH-generating enzyme of the branched chain amino acid degradation pathway. For total leaf chlorophyll content the mapping of significant SNPs for total leaf chlorophyll content was onto chromosomes 1, 2, 3, 8, 9, 10 and 11 mostly. Efrati A. et al. [34] mapped chlorophyllase on to chromosomes 6 (CHLASE 1) and chromosome 9 (CHLASE 2) in tomato lines. Gene Solyc11g017345.1 annotated as F-box/FBD/LRR-repeat protein was identified. F-box proteins regulate diverse cellular processes, including cell cycle transition, transcriptional regulation and signal transduction, by playing roles in Skp1p-cullin-F-box protein (SCF) complexes or non-SCF complexes [35]. At least 43 F-box protein-encoding genes have been found to be differentially expressed in rice seedlings subjected to different abiotic stress conditions [36]. It was also colocalized for the trait fruit shape index indicating that if one trait is introgressed other one will simultaneously improve which are key for achieving resilience to cold stress tolerance. Also lycopene is one of the most important carotenoid contributing for 98% of the pigment of tomato fruit colour. In rice seedlings [37] reported that carotenogenic gene mutations lead to increased oxidative stress in rice seedlings and verified the anti-oxidative function of carotenoids. The mapping of significant SNPs for lycopene was onto chromosomes 2, 5, 6, 8, 9, 10 and 11 mostly. Zhang J. et al. [38] in tomato detected significant marker trait associations for carotenoid-derived volatiles on chromosomes 6, 9 and 11 explaining > 10% phenotypic variation. Gene named Thioredoxin was identified. da Fonseca-Pereira P. et al. [39] reported that in Arabidopsis, Thioredoxin transcripts were more highly expressed under drought and produce an effect that is stronger during repetitive drought/recovery events.

Apart from reduced content of few compound’s there are certain compounds which accumulate in response to cold stress in tolerant plants and some which act as protectants. One of the most important amino acid known to impart stress tolerance is proline. A link between stress tolerance acquisition and proline accumulation has been established by [40]. Highly significant marker trait associations were discovered for proline content. For proline content the mapping of significant SNPs was onto chromosome 1, 2, 8 and 11 mostly. Sauvage C. et al. [41] also reported significant association for proline content on chromosome 2 in tomato. Similarly chilling-induced oxidative damage in tomatoes was reported to be mitigated by ascorbic acid [42]. It works by reducing electrolyte loss, lipid peroxidation and hydrogen peroxide levels. For ascorbic acid the mapping of significant SNPs was mostly onto chromosomes 2, 3, 9, 10, 11 and 12 mostly. Stevens R. et al. [43] identified common regions controlling ascorbic acid content on chromosomes, 9 and 10 in tomato and on chromosomes 9 and 11, [41] discovered significant associations for ascorbic acid content in tomato. Also, [44] in their research linked one locus to a previously identified ascorbic acid content large-effect QTL on chromosome 9 by [43] in tomato. Significant marker trait associations were detected on chromosome 3, 11 in tomato by [45]. Gene Solyc03g007550.4 annotated as LIM domain protein was identified for the trait. Park CJ et al. [46] reported that LIM genes have been involved in resistance against biotic and abiotic stresses in *Brassica.* It was also colocalized for the traits viz., number of fruits per plant thereby indicating an important correlation with fruit yield improvement under cold stressed conditions. Also colocalization with the traits viz., number of fruits per truss, number of fruits per plant, fruit yield per plant under normal and protected conditions indicates that simultaneous improvement can be done for many traits.

Compounds such as phenols and sugars protect plant cells against damage induced by cold stress in a variety of ways, including functioning as osmoprotectants, nutrients and interacting with the lipid bilayer [47]. Phenolic substances which are non-enzymatic antioxidants (Phenolic acids and flavonoids) can trap and scavange reactive oxygen species (ROS). For total phenols the mapping of significant SNPs was onto chromosome 1, 2, 3, 6, 7, 8, 9, 10 and 11 mostly. Ruggieri V. et al. [48] reported that phenol content was associated with markers on chromosomes 8 and chromosome 11 in tomato. The mapping of significant SNPs for total soluble sugars was onto chromosomes 1,2,3,6, 8, 9 and 11 mostly. Similar significant marker trait associations for soluble solids on chromosomes 2, 8 and 9 in tomato were detected by [45, 49] also detected significant associations for soluble solids on chromosome 2 in tomato. Significant marker trait associations for sugars on chromosomes 1, 2, 3 and 11 in tomato by were detected by [50]. Significant associations for soluble solid content on chromosomes 2, 8, 9 and 11 were detected by [50]. Sauvage C. et al. [41] also reported significant associations for sucrose content on chromosomes 2 and rhamnose on chromosome 8 in tomato.

To study yield and yield related traits in response to cold stress is very important aspect of the study because yield is the ultimate goal of a breeder along with climate resilience. For seedling length at transplant the mapping of significant SNPs was onto chromosomes 4, 5,6,8,9 and 11 mostly. Similar results were also observed by [51] in tomato mapped QTLs on chromosomes 1, 4, 6, 9 and 11 for various seedling traits viz., for hypocotyl length on chromosome 6, shoot weight on chromosome 9 and total root size on chromosome 9 and 11. For number of days to first harvest the mapping of significant SNPs was onto chromosomes 1, 2, 3, 8, 9 and 11 mostly. Significant association was observed by [52] in tomato for number of days between planting and ripening date of first fruit on chromosome 2 and for number of days between flowering and red ripe stage on chromosome 2. Significant marker trait associations in tomato for time to ripen on chromosomes 1, 2, 3, 9 and 10 were also observed by [50] in tomato.

For number of flowers per truss the mapping of significant SNPs was onto chromosome 3, 8, 9, 11 and 12 mostly. Significant associations were mapped for flower number on the third inflorescence on chromosome 9 by [44] in tomato. Also another important gene Solyc11g039880.2 annotated as Nucleoporin was identified. Dong CH et al. [53] reported that Nucleoporin (AtNUP160) in Arabidopsis was critical for the nucleocytoplasmic transport of mRNAs and it played important roles in plant growth and flowering time regulation and is required for cold stress tolerance. A defect in Nup160/SAR1 was also found to sensitize plants to chilling stress and disrupt acquired freezing tolerance. This gene is important in context of flowering under cold stressed conditions. For number of fruits per truss the mapping of significant SNPs was onto chromosome 1, 3, 7, 9, 11 and 12 mostly. Significant associations were mapped for fruit number on the third truss on chromosome 9 by [44] in tomato. Solyc11g150146.1 annotated as Elongation factor 1-alpha was identified and it was colocalized for the traits seedling vigor index and duration of harvest.

For fruit yield per plant the mapping of significant SNPs was onto chromosomes 1, 3, 4, 6, 7, 8, 9, 10, 11 and 12 mostly. Phan NT et al. [54] reported significant marker trait associations for fruit height on chromosomes 1, 3, 4, 7, 8; for fruit width on chromosomes 1, 3, 6, 10 and 11and for fruit weight on chromosomes 1, 3, 4, 6, 10 and 11 in tomato. For average fruit weight the mapping of significant SNPs was onto chromosomes 7, 8, 9 and 11 mostly. Significant associations were found for fruit weight on chromosome 11 by [49] and [45] in tomato while on chromosomes 7 and 9 by [52]. Candidate genes Solyc07g065840.2 annotated as molecular chaperone Hsp90-2 was identified it was also colocalized for the trait fruit yield per plant. Park CJ et al. [46] reported that heat shock proteins (HSPs) functioning as molecular chaperones are the key components responsible for protein folding, assembly, translocation and degradation under stress conditions and in many normal cellular processes. Shirasu K. et al. [55] also reported that HSP90s also play essential roles in plant immunity. Fruit weight is one the most important traits contributing to fruit yield and colocalization indicates a higher scope of improvement of fruit yield. Also in case of number of fruits the mapping of significant SNPs was onto chromosomes 2, 3, 9, 10, 11 and 12 mostly. Significant marker trait associations for fruit number on chromosomes 2, 10, 11 and 12 were reported by [50] in tomato. For fruit shape index the mapping of significant SNPs was onto chromosomes 1, 2, 3, 4, 6, 8, 9 and 11 mostly. Significant associations were found for fruit shape on chromosome 1, 8 and 9 by [45], on chromosome 8 by [49] and on chromosome 1, 2, 3, 4, 6, 8, 9 and 11 by [56] in tomato.

## Conclusion

A total of 4517 significant marker trait associations were revealed for cold tolerance traits which hampers the growth and development of the crop throughout the season. Also total of 5727 significant marker trait associations were revealed for yield and yield related traits uncovering important associations for fruit yield and directly contributing traits. 685 candidate genes were identified among all traits under study. Based on functional categorization, 60 genes were found to be associated with biological processes in these genomic regions. 7 among 60 were directly found to be related to abiotic stress tolerance and function directly or indirectly as stress responsive genes. This study could be used for breeding programme for developing high yielding hybrids.

### Supplementary Information

**Additional file 1.**

## Data Availability

The datasets generated and/or analysed during the current study are available in the NCBI repository. https://www.ncbi.nlm.nih.gov/geo/query/acc.cgi?acc=GSE200034.
