# Supplementary material for: Mapping phenotypic performance and novel SNPs for cold tolerance in tomato (Solanum lycopersicum) genotypes through GWAS and population genetics
Source: BMC Genom Data. 2024 Jan 27;25:9. doi: 10.1186/s12863-024-01190-5 (PMC10822167; doi:10.1186/s12863-024-01190-5)
Supplement: Supplementary file 1 — Additional file 1. [file 12863_2024_1190_MOESM1_ESM.docx]

**Table S1: Environment wise estimates of mean and range for different traits in tomato (*Solanum* spp.)**

|  | **ENV** | **DTE** | **SL(cm)** | **NOFPS** | **NODTFS** | **NOFPT** | **NODTFH** | **AFW (g)** | **FSI (cm^2^)** | **NOFPP** | **FYP (kg)** |
| --- | --- | --- | --- | --- | --- | --- | --- | --- | --- | --- | --- |
| Mean | E1 | 8.73 | 17.6 | 4.86 | 37.3 | 4.04 | 91.9 | 38.3 | 0.94 | 52.7 | 1.08 |
|  | E2 | 11.00 | 18.7 | 6.91 | 26.0 | 4.93 | 81.7 | 56.9 | 0.92 | 47.5 | 1.56 |
|  | E3 | 11.70 | 18.2 | 4.82 | 47.9 | 3.87 | 94.3 | 36.4 | 1.01 | 57.0 | 1.06 |
|  | E4 | 7.99 | 16.9 | 4.68 | 36.7 | 3.96 | 91.3 | 32.4 | 0.94 | 72.8 | 1.30 |
|  | E5 | 7.46 | 18.1 | 5.76 | 48.6 | 4.67 | 85.8 | 33.9 | 0.95 | 31.9 | 0.93 |
|  | E6 | 11.40 | 18.8 | 6.82 | 25.2 | 5.18 | 80.2 | 55.3 | 0.93 | 44.5 | 1.75 |
| Range | E1 | 5.50-16.40 | 7.33-25.94 | 2.66-12.73 | 24.74-54.33 | 2.33-9.00 | 86.00-95.66 | 0.95-127.46 | 0.53-1.46 | 3.33-210.66 | 0.19-4.14 |
|  | E2 | 7.66-14.66 | 7.66-28.00 | 3.33-25.66 | 22.66-47.66 | 2.33-17.33 | 81.66-91.66 | 2.45-318.00 | 0.50-1.25 | 4.00-434.66 | 0.03-4.90 |
|  | E3 | 10.33-15.66 | 7.55-27.49 | 2.33-14.66 | 36.66-89.33 | 1.66-9.00 | 83.66-134.33 | 0.96-162.19 | 0.58-1.62 | 5.66-357.33 | 0.14-3.33 |
|  | E4 | 5.00-14.66 | 6.28-23.78 | 2.33-19.66 | 27.33-54.33 | 2.00-17.66 | 85.66-96.66 | 1.32-148.49 | 0.47-2.17 | 4.66-364.66 | 0.19-3.98 |
|  | E5 | 4.66-9.33 | 7.14-24.98 | 2.00-20.33 | 33.00-83.00 | 1.66-18.33 | 81.33-96.33 | 0.87-120.09 | 0.65-1.45 | 3.00-335.00 | 0.06-2.11 |
|  | E6 | 9.66-15.66 | 6.77-24.52 | 3.66-24.66 | 22.33-43.33 | 3.33-18.33 | 81.66-87.66 | 1.42-319.89 | 0.51-1.41 | 3.20-402.33 | 0.08-5.73 |

|  | **ENV** | **NPP** | **PH (cm)** | **DOH** | **PV (%)** | **MDA (nmol/gfw)** | **PC (µmol/g)** | **TCC (mg/100g)** | **AA (mg/100g)** | **LYC (mg/100g)** | **TP (mg/100g)** | **TSS (mg/100g)** |
| --- | --- | --- | --- | --- | --- | --- | --- | --- | --- | --- | --- | --- |
| Mean | E1 | 3.50 | 118.57 | 113.48 | 47.9 | 6.43 | 0.68 | 13.6 | 15.3 | 6.41 | 1595.16 | 1.34 |
|  | E2 | 4.33 | 191.30 | 196.28 | 58.0 | 6.93 | 0.80 | 19.2 | 17.1 | 8.65 | 2245.19 | 1.07 |
|  | E3 | 3.29 | 94.4 | 140.10 | 49.5 | 6.80 | 0.70 | 12.1 | 15.7 | 6.71 | 1730.15 | 1.25 |
|  | E4 | 2.95 | 90.7 | 111.58 | 47.8 | 6.78 | 0.67 | 13.9 | 15.5 | 6.63 | 1651.14 | 1.39 |
|  | E5 | 2.52 | 87.0 | 95.5 | 47.8 | 5.99 | 0.67 | 9.63 | 14.7 | 4.63 | 1718.22 | 1.38 |
|  | E6 | 4.36 | 187.27 | 195.27 | 56.7 | 6.84 | 0.84 | 18.4 | 17.0 | 8.64 | 2217.19 | 1.02 |
| Range | E1 | 2.00-5.33 | 33.33-198.33 | 94.88-133.33 | 27.11-92.53 | 2.03-17.00 | 0.49-0.89 | 4.22-41.00 | 7.22-43.22 | 0.89-13.15 | 154.23-3382.70 | 0.41-2.91 |
|  | E2 | 2.33-10.33 | 48.63-397.00 | 160.66-217.66 | 14.61-93.03 | 1.11-13.11 | 0.32-1.24 | 7.19-47.22 | 7.17-45.17 | 0.81-14.17 | 871-4122.00 | 0.76-1.36 |
|  | E3 | 1.66-8.33 | 42.83-165.05 | 115.66-167.66 | 18.23-92.00 | 0.76-17.41 | 0.44-1.49 | 3.22-36.10 | 7.77-41.27 | 0.01-13.70 | 507.20-3893.00 | 0.78-1.99 |
|  | E4 | 1.66-5.66 | 48.18-137.33 | 92.66-133.33 | 22.21-93.84 | 0.12-16.00 | 0.51-0.87 | 3.23-40.97 | 7.54-40.14 | 0.88-16.70 | 151.20-3255.20 | 0.44-2.94 |
|  | E5 | 1.33-4.00 | 42.15-156.92 | 85.66-120.33 | 10.13-92.67 | 0.26-17.16 | 0.49-0.89 | 3.22-27.22 | 6.19-42.17 | 0.41-10.22 | 211.20-3407.20 | 0.77-2.00 |
|  | E6 | 2.66-9.00 | 53.35-394.16 | 172-214.66 | 13.43-95.25 | 2.06-13.22 | 0.30-1.22 | 5.72-54.50 | 7.88-46.22 | 0.79-16.24 | 451-4050.00 | 0.78-1.39 |

Note: DTE: Days to emergence, SL: Seedling length at transplant, NOFPS: Number of flowers per truss, NODTFS: Number of days to first fruit set, NOFPT: Number of fruits per truss, NODTFH: Number of days to first harvest, A FW: Average fruit weight, FSI: Fruit shape index, NOFPP: Number of fruits per plant FYP: Fruit yield per plant, NPP: Number of primary branches, PH: Plant height, DOH: Duration of harvest, PV: Pollen viability, MDA: Malondialdehyde, PC: Proline content, TCC: Total leaf chlorophyll content, AA: Ascorbic acid, LYC: Lycopene content, TP: Total phenols, TSS: Total soluble sugars,

Note: E1: Field, normal sown (2019); E2: Polyhouse sown (2019); E3: Field, early sown (2020); E4: Field, normal sown (2020); E5: Field, late sown (2020); E6: Polyhouse sown (2020)
